# Atlantic Oceanic Squids in the “Grey Speciation Zone”

**DOI:** 10.1093/icb/icad116

**Published:** 2023-08-21

**Authors:** Fernando Á Fernández-Álvarez, Gustavo Sanchez, Diego Deville, Morag Taite, Roger Villanueva, A Louise Allcock

**Affiliations:** Institut de Ciències del Mar (ICM), CSIC, Passeig Marítim de la Barceloneta 37–49, 08003 Barcelona, Spain; Molecular Genetics Unit, Okinawa Institute of Science and Technology, Onna, Okinawa 904-0412, Japan; Graduate School of Integrated Sciences for Life, Hiroshima University, Higashihiroshima, Hiroshima 739-8528, Japan; Ryan Institute and School of Natural Sciences, University of Galway, University Road, Galway H91 TK33, Ireland; Institut de Ciències del Mar (ICM), CSIC, Passeig Marítim de la Barceloneta 37–49, 08003 Barcelona, Spain; Ryan Institute and School of Natural Sciences, University of Galway, University Road, Galway H91 TK33, Ireland

## Abstract

Cryptic species complexes represent an important challenge for the adequate characterization of Earth’s biodiversity. Oceanic organisms tend to have greater unrecognized cryptic biodiversity since the marine realm was often considered to lack hard barriers to genetic exchange. Here, we tested the effect of several Atlantic and Mediterranean oceanic barriers on 16 morphospecies of oceanic squids of the orders Oegopsida and Bathyteuthida using three mitochondrial and one nuclear molecular marker and five species delimitation methods. Number of species recognized within each morphospecies differed among different markers and analyses, but we found strong evidence of cryptic biodiversity in at least four of the studied species (*Chtenopteryx sicula, Chtenopteryx canariensis, Ancistrocheirus lesueurii*, and *Galiteuthis armata*). There were highly geographically structured units within *Helicocranchia navossae* that could either represent recently diverged species or population structure. Although the species studied here can be considered relatively passive with respect to oceanic currents, cryptic speciation patterns showed few signs of being related to oceanic currents. We hypothesize that the bathymetry of the egg masses and duration of the paralarval stage might influence the geographic distribution of oceanic squids. Because the results of different markers and different species delimitation methods are inconsistent and because molecular data encompassing broad geographic sampling areas for oceanic squids are scarce and finding morphological diagnostic characters for early life stages is difficult, it is challenging to assess the species boundaries for many of these species. Thus, we consider many to be in the “grey speciation zone.” As many oceanic squids have cosmopolitan distributions, new studies combining genomic and morphological information from specimens collected worldwide are needed to correctly assess the actual oceanic squid biodiversity.

## Introduction

One important challenge to ensure Earth’s biodiversity is fully characterized is to correctly delimit cryptic species complexes (Fišer et al. 2018). This is particularly relevant now, in the time of the Sixth Mass Extinction (Barnosky et al. 2011), to ensure we understand the true extent of the biodiversity crisis. Since marine environments were traditionally regarded as devoid of physical barriers to gene flow, animals spread across large geographic areas were commonly regarded as conspecifics, sometimes even when morphological differences were already known (Fernández-Álvarez et al. 2020). Despite this misconception, marine currents and other oceanic phenomena can constitute effective barriers against faunal movements, larval dispersion, and, in summary, the gene flow of populations with wide distributions (Pascual et al. 2017). These oceanic barriers result in major pelagic provinces, as described by Spalding et al. (2012). If an oceanic barrier lasts long enough, it can lead to speciation phenomena. If similar selective pressures affect the morphology of the recently diverged species, those species may have similar morphological features, difficult or impossible to differentiate using only morphological cues.

The Atlantic Ocean offers many such barriers, for example, the North and South Atlantic oceanic currents, the Canary Current, the Gulf Stream, and the Benguela and Brazil currents (Talley et al. 2011); the Mediterranean Sea also displays a large number of barriers, with the Strait of Gibraltar, the Almeria-Oran Front, the Ibiza Channel, and the Messina Strait among the most important (Pascual et al. 2017). All these barriers could potentially facilitate cryptic species assemblages of well-known pelagic morphospecies. Due to a paucity of hard structures in cephalopod bodies, this group is a good candidate to harbor cryptic biodiversity. Interestingly, many cephalopod species complexes have been reported previously in both coastal (Allcock et al. 2011; Anderson et al. 2011; Sales et al. 2017) and oceanic cephalopods (Staaf et al. 2010; Escánez et al. 2018; Fernández-Álvarez et al. 2020, 2021). However, the cryptic biodiversity of epi-, meso-, and bathypelagic cephalopods of the orders Oegopsida and Bathyteuthida remains largely unexplored due to the complications that their oceanic lifestyle poses for scientific collection and identification, in particular the overrepresentation of young life stages in collections, which are commonly difficult or impossible to identify based on morphological characteristics (Sweeney et al. 1992).

Most studies focused on cryptic biodiversity assessment rely on traditional Sanger single-marker sequencing methodologies, such as DNA barcoding (Hebert et al. 2003). Indeed, *cytochrome c subunit 1* (*cox1*) is the marker of choice for metazoan DNA barcoding, and an extensive database of comparator sequences for this marker exists. Other potential barcodes include those developed for eDNA (since such markers have been developed specifically to identify species); in cephalopods, these include mitochondrial 12S rRNA (Sanchez and Fernández-Álvarez 2021) and nuclear 18S rRNA (de Jonge et al. 2021). Although nuclear ribosomal genes such as 18S rRNA are traditionally considered conserved, evidence of high substitution rates in cephalopods has previously been reported (Meyer et al. 2010). Genome skimming (Dodsworth 2015), a shallow whole genome sequencing method that allows high copy regions of the genome, such as the complete mitogenome and the complete nuclear ribosomal cluster, to be obtained, critically captures the standard *cox1* barcode, both proposed eDNA markers, and also allows the overall number of markers to be increased. Despite its advantages, such as its relatively low price and compatibility with low-quality DNA samples (Abalde et al. 2023), there are only a few works exploring this method for cephalopods (Sanchez et al. 2021; Fernández-Álvarez et al. 2022; Sánchez-Márquez et al. 2023; Taite et al. 2023a).

Here, we assess cryptic diversity in oceanic squids by comparing five species delimitation techniques to data generated from markers that have potentially taken different evolutionary pathways (nuclear genome versus mitochondrial genome) and that have evolved at different rates.

## Material and methods

### Samples

Oceanic squids were mainly collected during the oceanic cruises MAFIA (Olivar et al. 2017), BATHYPELAGIC, and SUMMER (Olivar et al. 2022), which sampled international waters from east of Brazil to south of Iceland in the Atlantic and in the Spanish EEZ in the NE Atlantic and Western Mediterranean (see Supplementary Fig. S1). Nominal species distributed on both sides of one or more oceanic barriers were selected for this study, including individuals identified based on morphology as 16 different species: *Chtenopteryx sicula* (Vérany, 1851), *Chtenopteryx canariensis* Salcedo-Vargas & Guerrero-Kommritz, 2000, *Ancistrocheirus lesueurii* (d'Orbigny, 1842), *Leachia atlantica* (Degner, 1925), *Liocranchia reinhardti* (Steenstrup, 1856), *Bathothauma lyromma* Chun, 1906, *Egea inermis* Joubin, 1933, *Teuthowenia megalops* (Prosch, 1849), *Teuthowenia maculata* (Leach, 1817), *Helicocranchia navossae* Judkins, Rose-Mann, Lindgren, Taite, S. Bush & Vecchione, 2022, *Galiteuthis armata* Joubin, 1898, *Abraliopsis morisii* (Vérany, 1839), *Grimalditeuthis bonplandi* (Vérany, 1839), *Mastigoteuthis agassizii* A. E. Verrill, 1881, *Pyroteuthis margaritifera* (Rüppell, 1844), and *Pterygioteuthis gemmata* Chun, 1908. For convenience, divergent clades among these morphospecies received different names (see “nomenclature” in the Supplementary Material). A small piece of mantle tissue was fixed in ethanol for DNA extraction, and the remaining body was fixed in formalin and vouchered at the Marine Biological Reference Collections (CBMR-ICM), Barcelona (Guerrero et al. 2020) (see Supplementary Table S1).

### DNA extraction, sequencing, and bioinformatics

DNA was extracted using the Purelink genomic DNA Mini kit (Invitrogen, MA, USA) following the manufacturer’s instructions. A preliminary study on the biodiversity of oegopsid and bathyteuthid squids in Atlantic waters was undertaken using DNA barcoding methodologies described by Fernández-Álvarez et al. (2021; see Supplementary Material for more details). A small fragment of the small ribosomal mitochondrial unit (12S rRNA) was sequenced using new primers that amplify a variable region potentially suitable for species identification using eDNA (Sanchez and Fernández-Álvarez 2021; see Supplementary Material for more details). Selected specimens were also sequenced through genome skimming (Dodsworth 2015), roughly following the methodologies described by Sánchez-Márquez et al. (2023; see Supplementary Material for more details). This allowed the complete mitogenome and the small nuclear ribosomal unit (18S rRNA) to be assembled, such that species delimitation could be investigated across four marker databases (two previously reported as suitable for eDNA). When available, sequences of members of the same morphospecies and genus of each molecular marker except 12S rRNA, where we experienced alignment difficulties within a hypervariable region, were retrieved from GenBank (see Supplementary Table S1), resulting in four different databases based on: (1) the c*ytochrome c subunit 1 database* (cox1); (2) 12S rRNA (12S); (3) mitochondrial protein-coding genes (mitoPCG); and (4) 18S rRNA (18S). The nomenclature of each lineage was mainly based on the identified clades from *cox1*, as this dataset included a larger number of lineages (Supplementary Table S1).

### Phylogenetic and species delimitation analyses

The databases *cox1* and mitoPCG were manually aligned, while 12S and 18S were aligned using the MAFFT server (https://mafft.cbrc.jp/alignment/server/, Katoh et al. 2009) with the Q-INS-I iterative refinement method. Phylogenetic analyses of maximum likelihood (ML) and Bayesian inference (BI) coalescent analyses were performed in IQTREE (Nguyen et al. 2015; Hoang et al. 2018) and BEAST 2.6.4 (Bouckaert et al. 2019), respectively. Five species delimitation methods were compared for each matrix: Poisson tree processes (PTP; Zhang et al. 2013), Assemble Species by Automatic Partitioning (ASAP; Puillandre et al. 2020), TCS haplotype networks (Clement et al. 2000), the Generalized Mixed Yule Coalescent (GMYC; Pons et al. 2006), and a Bayesian implementation of GMYC (Reid and Carstens 2012). Empirical studies show that TCS haplotype networks with a maximal connectivity limit of 95% commonly reflect species assemblages (e.g., Pons et al. 2006; Hart and Sunday 2007; Kang et al. 2015; Taite et al. 2023b). See Supplementary Material and methods for technical aspects of the phylogenetic and species delimitation analyses. In this work, the unified species concept (De Queiroz 2007) was applied. The concordance of results of molecular species delimitation methods was considered as confirmation of the reproductive isolation between groups and therefore the presence of cryptic biodiversity. In the same way, contradictory results in the number of delimited species were considered as indicative that these lineages are on the boundary between large population structure and early speciation, defined here as the “grey speciation zone,” where the species taxonomy can be considered controversial (Roux et al. 2016). Uncorrected genetic distances (*p*-distances) between and within cryptic species were calculated with MEGA 11 (Tamura et al. 2021). The specific labels employed here were defined according to the clusters identified in Supplementary Table S1.

Maps showing the geographic location of sequenced samples with geographic information available were built using QGIS v.3.10 (QGIS 2020). Within each map, species were represented with different symbols and colors to ease visualization.

## Results

### Molecular species delimitation

**Fig. 1:**
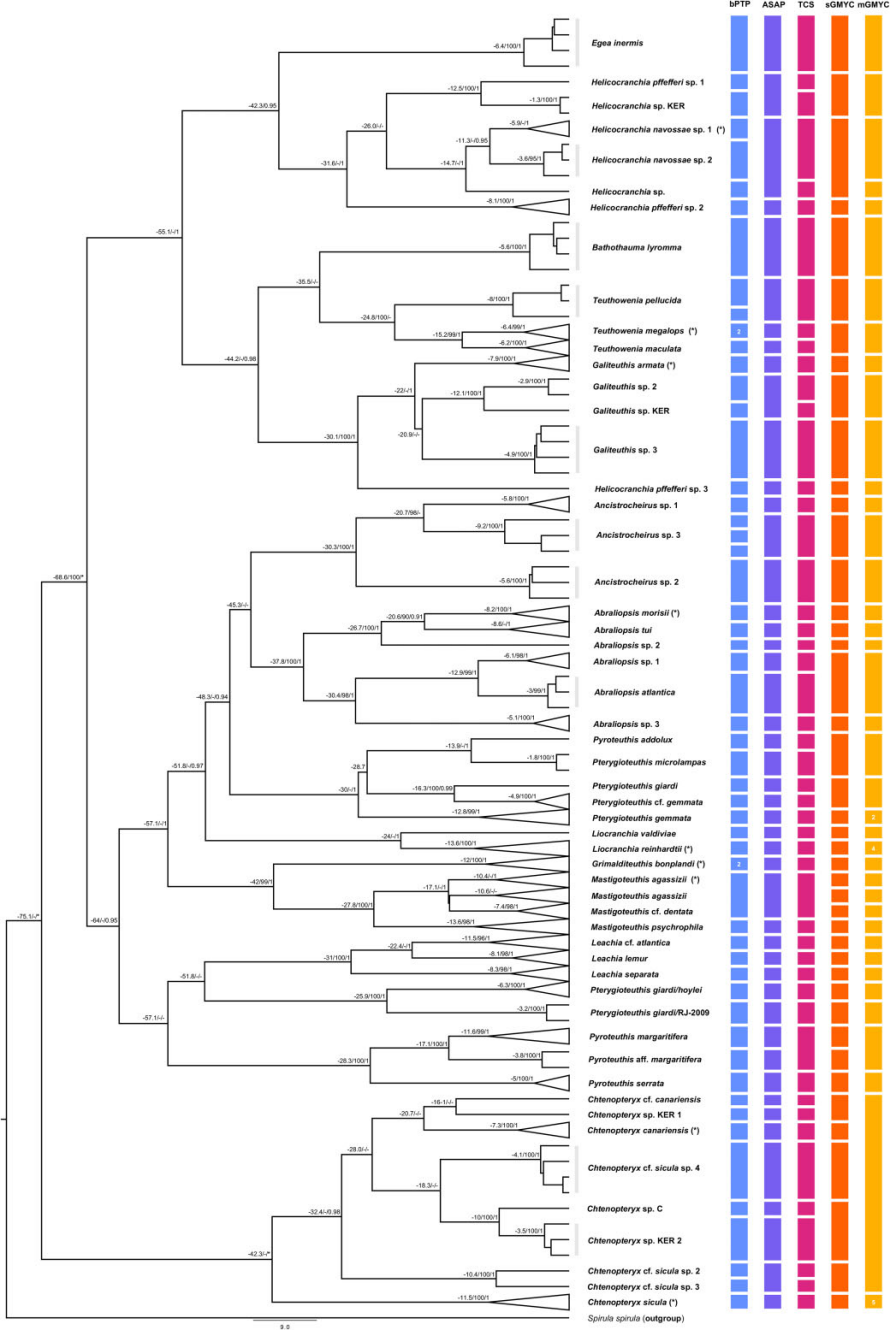
Summary of species delimitation analyses obtained with the *cox1* database, depicted over the coalescent analyses obtained through BEAST 2.6.4 (Bouckaert et al. 2019). For convenience, clades formed by six or more sequences were collapsed. Numbers above branches refer to the node ages (Mya) and to the support of the node as the ultrafast bootstrap percentages (%) from the ML analysis and the posterior probabilities from the coalescent analysis, respectively. Bootstrap and posterior probabilities of <90% and 0.9, respectively, were not recorded. Dashes indicate a lack of support or clades with a different topology in the ML analysis. Asterisks designate clades that were set up as monophyletic for the coalescent analysis. Asterisks inside parenthesis designate lineages with one or more sequences coming from a locality close to the type locality of a nominal species. Vertical lines summarized the results from bPTP, ASAP, TCS, sGMYC, and mGMYC (see Supplementary Material and methods for more details). Numbers inside some results for delimitation analysis summarized the number of species found for a collapsed clade.

The four databases had different coverage in both the number of species and specimens and the geographic span. While *cox1* and 12S Sanger sequencing were fairly successful and most studied specimens are present in both databases, there are only 12S sequences for *Pterygioteuthis* sp. 2 (Supplementary Table S1). Genome skimming was less successful: the library preparation failed for *T. maculata, Le*. cf. *atlantica*, one of the two specimens of *Li. reinhardtii, Galiteuthis* sp. 2, *Abraliopsis* sp. 1, *Gr. bonplandi*, and two specimens of *Pt. gemmata*. Therefore, these species lack mitoPCG and/or 18S. The most complete database was *cox1*, including 28 nominal species and 13 additional clades formed by sequences identified under other taxonomic labels (e.g., *Pyroteuthis* RJ-2009; see Supplementary Table S1). Remaining databases ranged from 15 to 21 nominal species (see Supplementary Results). For a complete picture of the results of all species delimitation analyses, see Supplementary Results and Figs. 1 and S2–8. The differences in taxonomic coverage make comparisons across different databases difficult. Unsurprisingly, the most conservative database was 18S, where four out of five species delimitation analyses recognized fewer than the number of included nominal species, with some genera recognized as a single species (*Chtenopteryx, Leachia*, and *Teuthowenia*, Supplementary Fig. S4). The mean number of species identified by delimitation methods in the three mitochondrial databases was higher than the number of nominal species included: species delimitation recognized on average 30, 38, and 33% more species than the nominal species count in the *cox1*, 12S, and mitoPCG databases, respectively.

For a brief summary of the number of species recognized among the focal Atlantic species in the four databases, see Table 1. For *C. sicula*, up to four cryptic species are recognized: one in the North Atlantic and Mediterranean; three in the North Pacific. The South Atlantic specimen of *C*. cf. *canariensis* was consistently recognized as a different species to other *C. canariensis* in all mitochondrial databases. 18S data were inconclusive for this genus as different delimitation methods gave different results (Supplementary Fig. S4). *Ancistrocheirus lesueurii* was recognized as three different species by *cox1* and mitoPCG analyses: two co-occurring in Atlantic waters and another in the North Pacific. The 12S analyses recognized two *An. lesueurii* species but lacked sequences from the North Pacific. 18S data were also inconclusive for *Ancistrocheirus. Liocranchia reinhardtii* and *E. inermis* were each consistently recognized as a single species across all the databases that had more than one representative sequence. *Leachia* cf. *atlantica* was also recognized as a single species by the *cox1* and 12S databases but clustered as conspecific with *Le. lemur* in the 18S database. All mitochondrial databases consistently recognized a single *B. lyromma*, but three out of five 18S analyses recovered it as two species. The three *Teuthowenia* species were each recognized as a single species in the *cox1* analyses, and *T. megalops* and *T. pellucida* were recognized as distinct species by most species delimitation methods in mitoPCG. In contrast, the ribosomal databases recovered the entire genus as conspecific (only *T. megalops* and *T. pellucida* were present in the 18S database). *Helicocranchia navossae* was consistently recognized as a single species by the ribosomal databases, but one and two of the five species delimitation analyses of the *cox1* and mitoPCG analyses, respectively, recovered *H. navossae* spp. 1 and 2 (as labeled in Supplementary Table S1) as different species. *Galiteuthis armata* was recognized as distinct from North Atlantic *Galiteuthis* sp. 2 (identified by morphology as *Ga. armata*) and South Atlantic *Galiteuthis* sp. 3 in the *cox1* database (Fig. 1). Different *cox1* analyses recovered New Zealand *Galiteuthis* sp. KER either as conspecific with or distinct from *Galiteuthis* sp. 2. The 12S database recovered *Ga. armata* and *Galiteuthis* sp. 2 either as conspecifics or as two different species, depending on the analysis. Individuals from the clades *Ab. morisii* and *Abraliopsis* spp. 1 and 2 were identified using morphology as *Ab. morisii*. While *Ab. morisii* and *Abraliopsis* sp. 2 were recognized as different species in the *cox1* analyses, they were not distinct species in the 12S analyses. *Abraliopsis* sp. 1 and *Ab. atlantica* were recovered as either conspecific or different species according to different analyses in the *cox1* database (Fig. 1), and a single divergent 12S haplotype was identified as a further species in several 12S species delimitation analyses (Supplementary Fig. S2). North and South Atlantic *Gr. bonplandi* were recognized as a single species in *cox1* analyses, but two of the five species delimitation analyses using the 12S database isolated the North Atlantic specimen from the two South Atlantic ones (Supplementary Fig. S2). North and South Atlantic *M. agassizii* were recognized as the same species in 12S and 18S analyses, but either the same species or two different species by the mitoPCG database. *Cox1* analyses recognized all *M. agassizii* and *M*. cf. *dentata* sequences as a single species, but both versions of GMYC recognized all *M*. cf. *dentata* and two *M. agassizii* clades, including North Atlantic and South Atlantic and North Atlantic and New Zealand sequences, as three different species. Two of five species delimitation analyses on the *cox1* database recognized North Atlantic *Py. margaritifera* as conspecific with New Zealand *Py*. aff. *margaritifera*. Remaining databases consistently recognize North Atlantic *Py. margaritifera* as a single species. North Atlantic *Pt. gemmata* and New Zealand *Pt*. cf. *gemmata* sequences were recovered as different species in all *cox1* species delimitation analyses, but the latter was recovered as conspecific to South Atlantic *Pt. giardi* GenBank sequence GU145065 by the GMYC analyses. Two highly divergent 12S sequences from two specimens identified by morphology as *Pt. margaritifera* from the North Atlantic (labeled as *Pterygioteuthis* sp. 2 in Supplementary Table S1) were recovered as an additional species in all species delimitation analyses. Unfortunately, *cox1* PCRs for these two specimens were unsuccessful.

**Table 1:** Summary of the main results based on the species delimitation methods of the four matrices for the 16 Atlantic morphospecies studied in this study

		Evidence of cryptic species				
Species	Bathymetric distribution	*cox1*	12S	mitoPCG	18S	Comments
*Chtenopteryx sicula* *	B, M, and E	3–4 [NA + Med*], [NP], [P]	1 [NA + Med*]	2 [NA + Med*], [NP]	Inconclusive	
*Chtenopteryx canariensis* *	B, M, and E	2 [NA* + SA], [SA]	2 [NA*] + [SA]	2 [NA*], [SA]	Inconclusive	
*Ancistrocheirus lesueurii*	M and E	3 [NA + Med], [NA], [NP]	2 [NA + Med], [NA]	3 [NA], [NA], [NP]	Inconclusive	Type locality of *An. lesueurii* is the Indian Ocean (Bello 1992), currently not sampled.
*Leachia atlantica*	B, M, and E	1 [NA]	1 [NA]	–	1 [NA + SA]	Conspecific with *Le. lemur* sequence only in 18S analyses
*Liocranchia reinhardti* *	B, M, and E	1 [NA]	1 [NA]	–	–	
*Bathothauma lyromma*	M and E	1 [NA]	1 [NA]	1 [NA]	1–2? [NA]	
*Egea inermis*	M and E	1 [NA + SA]	–	1 [NA + SA]	1 [NA + SA]	
Teuthowenia megalops *	M and E	1 [NA]	–	–	–	Genus *Teuthowenia* considered as a single species by 12S and 18S analyses
*Teuthowenia maculata*	M and E	1 [NA]	1 [NA]	–	–	
Helicocranchia navossae *	M and E	1 [NA]	1 [NA]	1–2? [NA*], [NA]	1 [NA]	Some *cox*1 species delimitation analyses consider *H. navossae* conspecific with the South Atlantic *Helicocranchia* sp. sequence KF369197
Galiteuthis armata *	M and E	3–4? [NA + Med*], [NA + SP?], [SP] [NA + SA]	1–2 [NA + Med*], [NA]	–	1 [NA + Med*]	
Abraliopsis morisii *	M and E	2–3? [NA + Med*], [NA], [NA]	2–3 [NA + Med*], [NA], [NA]	2 [NA + Med*], [NA]	1 [NA + Med*]	
Grimalditeuthis bonplandi *	B, M, and E	1 [NA* + SA]	1–2 [NA*], [SA]	–	1 [NA*]	
Mastigoteuthis agassizii *	B, M, and E	1–3? [NA* + SA], [NA], [SP]	1 [NA* + SA]	1–2? [NA*] [NA]	1 [NA* + SA]	
*Pyroteuthis margaritifera*	E	1–2 [NA], [SP]	1 [NA]	1 [NA]	1 [NA]	
*Pterygioteuthis gemmata*	E	2 [NA], [SP + SA?]	2 [NA], [NA]	–	–	*Pterygioteuthis* cf. *gemmata* was recovered as the same species as *Pt. giardi* sequence GU145065 in both *cox1* GMYC analyses. In the 12S matrix, *Pterygioteuthis* sp. 2, identified as *Pt. gemmata*, was recovered as a different species. Possible case of cryptic biodiversity.

Asterisks on the species name indicate that a specimen near the type locality of the nominal species was included in one of the clusters. For the bathymetric distribution all ontogenetic stages were considered. The total number of species under a taxonomic name for each marker is indicated by a number, and the geographic distribution of each taxon is indicated between brackets. The number of species is indicated with a range if four of the species delimitation methods returned these values, or with a question mark if 2–3 species delimitation methods point to that result. If only a single species delimitation method points to a particular result it is not reported here. Abbreviations: B, bathypelagic; M, mesopelagic; E, epipelagic; NA, North Atlantic; SA, South Atlantic; Med, Mediterranean; NP, North Pacific; SP, South Pacific; P, Pacific;—, not available.

The Bayesian GMYC outputs produced probability values for the different identified species (Supplementary Fig. S5–8). These values can be used as another source of evidence when there are differences in the number of recognized species by different methods (see Supplementary Results).

Complete analyses of the intra- and interspecific *p*-distances for each marker are provided in the Supplementary Results and Supplementary Tables S2–9. In summary, all datasets except mitoPCG showed an overlap among intra- and interspecific distances using the nomenclature defined for this work (Supplementary Material and methods and Supplementary Table S1), but it must be noted that only four species have intraspecific representation for that marker (Supplementary Table S6). In most cases, *cox1* intraspecific distances were below 1% (Supplementary Table S2), and larger intraspecific distances (up to 2.4%) were commonly associated with the recognition of more than a single species by species delimitation methods (Supplementary Results). Interspecific *cox1* distances ranged from 0.9 to 18.6% (Supplementary Table S3). The 12S showed the largest variation range at both intra- and interspecific levels (Supplementary Tables S4–5). The mitoPCG intraspecific distances were <0.9%, while interspecific distances ranged from 2 to 25.5% (Supplementary Tables S6–7). Most species showed no 18S variation (Supplementary Table S8), with the exceptions of *C. sicula* (0.07%) and *B. lyromma* (1.2%). Interspecific 18S distance range was 0–5.1%, but distances among related species were usually higher than 0.1.

### Geographic distribution

The distribution of each identified clade was plotted in relation to major oceanographic currents in order to elucidate whether there is any relationship among species boundaries and oceanographic features (Supplementary Figs. S9–20). When a species was present on both sides of an oceanographic barrier, then that barrier apparently does not interrupt gene flow sufficiently to induce an allopatric speciation event. Four different clades were identified as *C. sicula*, three of them in Pacific waters, but there were only enough samples to make geographic inferences for the Atlantic and Mediterranean. Highly divergent species identified as *C. canariensis* and *C*. cf. *canariensis* co-occur in the South Atlantic, and *C. canariensis* individuals are present on both sides of the Canary Current and the three Atlantic Equatorial Currents (Supplementary Fig. S9). Atlantic *Ancistrocheirus* sp. 1 is able to cross Mediterranean oceanic barriers (the Strait of Gibraltar, Almeria-Oran Front, and Ibiza Channel) and the Canary Current, while it seems that the Gulf Stream is not a barrier for *Ancistrocheirus* sp. 2 (Supplementary Fig. S10). The Atlantic oceanic currents do not seem to produce allopatric speciation events in *Le*. cf. *atlantica* (Supplementary Fig. S11), *Li. reindhardtii* (Supplementary Fig. S12), *B. lyromma, E. inermis* (Supplementary Fig. S13), *Ga. armata, Galiteuthis* sp. 2 (Supplementary Fig. S16), and *M. agassizii*, regardless of whether it is considered conspecific with *M*. cf. *dentata, Gr. bonplandi* (Supplementary Fig. S17), and *Pt. gemmata* (Supplementary Fig. S19). The Gulf Stream does not constitute a barrier for *Py. margaritifera* (Supplementary Fig. S20). On the contrary, the distribution of the morphologically different *T. megalops* and *T. maculata* seems to be determined by the Canary Current (Supplementary Fig. S14). In a similar way, *Helicocranchia* clades seem to be influenced by the main Atlantic currents, as both *H. navossae* spp. 1 and 2 and *Helicocranchia* sp. clades are separated by the Atlantic Equatorial Currents and likely the Canary Current (Supplementary Fig. S15). The Gulf Stream does not seem to create a barrier among *H. navossae* sp. 1 specimens. Despite the clear allopatric geographic structure observed for the three *Helicocranchia* clades, many species delimitation methods consider these three clades as monospecific. Due to a lack of samples, it is not possible to assess the degree of overlapping distributions among *Ab. morisii* and *Abraliopsis* spp. 1 and 2, but at least *Ab. morisii* and *Abraliopsis* sp. 2 are sympatric north of the Canary Current (Supplementary Fig. S18). The Mediterranean oceanographic barriers are not identified as a barrier for genetic exchange among *Ab. morisii* individuals.

## Discussion

### Cryptic biodiversity

We tested the effect of several Atlantic and Mediterranean oceanic barriers on 16 morphospecies of oceanic squids of the orders Oegopsida and Bathyteuthida using three mitochondrial and one nuclear molecular marker and five species delimitation methods. The number of species recognized within each morphospecies differed among different markers and analyses. Strong evidence of cryptic biodiversity was found in at least four of the studied species (*C. sicula, C. canariensis, An. lesueurii*, and *Ga. armata*). Additionally, *H. navossae* showed highly geographically structured units that could either represent recently diverged species or population structure.

Only in *E. inermis* were the results consistent for all analyses, although in *H. novossae* and *Gr. bonplandi*, similar numbers of species were identified (Table 1). In general terms, nuclear 18S rRNA data were too conserved to identify species, and, in many cases, different well-defined and accepted species were clustered as a single species with this marker (e.g., *Chtenopteryx* or *Leachia* spp., three out of four *Pterygioteuthis* spp., Supplementary Fig. S4), rendering it not very useful for species delimitation. This came as no surprise, as nuclear ribosomal genes tend to have very conserved regions used to resolve deep nodes in cephalopod phylogenies (Allcock et al. 2015). However, the discovery of large divergences among the 18S sequences of the two *B. lyromma* specimens, which were interpreted as different species by three of the five species delimitation methods, is particularly interesting, as it could suggest that cryptic species are hidden in the remaining genes, perhaps by mitochondrial introgression. Although cephalopod cryptic biodiversity studies frequently use mitochondrial markers (e.g., Staaf et al. 2010; Allcock et al. 2011; Cheng et al. 2014; Fernández-Álvarez et al. 2020, 2021), recent hybridization among recently speciated taxa is a possibility that nuclear markers might solve. Unfortunately, our results indicate that species-level inferences are beyond the resolution of 18S and faster-evolving nuclear genes are more suitable when assessing potential introgression. These results are also important for assessing the availability of this marker for eDNA, as specific primers for this gene have been developed (de Jonge et al. 2021) and used (Visser et al. 2021), but to the best of our knowledge, studies focusing on the suitability of this marker for species-level identifications are lacking.

Species delimitation methods applied to the mitochondrial databases revealed 30–37% cryptic species in addition to the number of nominal species in each database. In the particular case of *Teuthowenia*, the three accepted and morphologically different species were clustered within a single species by 12S (Supplementary Fig. S2). Thus, it seems that 12S is not appropriate to correctly assess the biodiversity within this genus. While no further different congeneric morphospecies were clustered together in the same way, this marker did not find cryptic biodiversity among *Ga. armata* and *Ab. morisii*, which was strongly supported by both *cox1* (Fig. 1) and mitoPCG (Supplementary Fig. S3). Highly divergent 12S sequences from two poorly preserved individuals identified using morphological characters as *Pt. gemmata* were recovered as a different species (*Pterygioteuthis* sp. 2) by all species delimitation methods in the 12S database. However, comparisons among this taxon and other *Pterygioteuthis* spp. are not possible as no other marker was obtained, and therefore misidentification of a previously described species cannot be ruled out. While the results of the mitoPCG and the *cox1* matrix were very similar among species present in both databases, *cox1* revealed more cryptic species because of the greater number of included sequences. This reflects the fact that *cox1* DNA barcodes have been sequenced for decades (e.g., Groenenberg et al. 2009; Allcock et al. 2011), and the production of complete mitogenomes is still limited at present with multiple mitogenomes sequenced for only a few species (e.g., the giant squid, Winkelmann et al. 2013). Furthermore, congeneric representation among mitogenomes is usually lacking since mitogenomes are widely used in attempts to resolve deeper relationships. Thus, our *cox1* database had greater geographic, taxonomic, and intraspecific representation. Both *cox1* and mitoPCG had more resolution than either 12S or 18S and were able to identify cryptic lineages in the oegopsid genera *Helicocranchia, Galiteuthis*, and *Abraliopsis*.

The new sequences of the bathyteuthid *C. sicula* include a Mediterranean individual. As the type locality of this species is Messina, Central Mediterranean (Sweeney 2017), we consider this species *C. sicula* s.s. and the other 2-3 Pacific clades as cryptic species. Two clades identified by morphology as *C. canariensis* are cryptic, so we consider the clade closest to the type locality (Canary Islands) as *C. canariensis* s.s. and *C*. cf. *canariensis* as an undescribed species. Currently, three *Chtenopteryx* species are accepted, although their taxonomy is very confusing (e.g., Escánez et al. 2018). Species delimitation analyses revealed at least four species in the mitoPCG database and nine species in the *cox1* database, which had higher taxon representation. A recent single study of the bathyteuthid Atlantic deep-sea squids doubled their known biodiversity from three to six species (Judkins et al. 2020). Hence, it is likely that the actual biodiversity of this order of oceanic squids is vastly underestimated.

This study supports the suggestion, previously made using both molecular data (Roura et al. 2019; Fernández-Álvarez et al. 2022) and the morphology of paralarvae (Sweeney et al. 1992), that the sharpear enope squid *An. lesueurii* actually comprises at least three highly divergent species, two co-occurring in North Atlantic waters and another in Pacific waters. Assessing which species actually represents the nominal species and which synonyms should be resurrected to name each clade is beyond the scope of this study, as no sequence from the type locality (Indian Ocean, fide Bello 1992) is currently available and the diversity of this clade across the world is largely unexplored.

Among glass squids, *Le*. cf. *atlantica, Li. reinhardti, E. inermis, T. megalops*, and *T. maculata* showed no clear signs of comprising cryptic species. However, the recently described *H. navossae* showed moderately divergent geographically structured clades that are potentially early stages of speciation; some species delimitation analyses considered this to be a single species, while others did not, and sometimes *Helicocranchia* sp. was included within the species (Fig. 1). These three lineages were geographically isolated by the North and South Atlantic Equatorial Currents (Supplementary Fig. S15). Although this variation could simply be population structure, it is important to point out that only one out of six species delimitation methods was able to reflect the biodiversity within related bobtails (Fernández-Álvarez et al. 2021) that diverged in very recent times (2.4 Mya, Sánchez et al. 2021). Similarly, Costa et al. (2021) found two morphologically different dart squid and Argus brief squid, which were recovered as the same species based on molecular data. Here, rapid morphological drift had occurred, but since open-water pelagic environments could produce very similar selective pressures on the morphology of related squids, molecular divergence after a recent speciation might not be accompanied by parallel morphological changes. This might explain why highly skilled cephalopod taxonomists failed to correctly allocate shortfin squids to their molecular species (Carlini et al. 2006) or why cryptic species are present in the neon (Fernández-Álvarez et al. 2020) and purpleback squids (Staaf et al. 2010; Jeena et al. 2023). Even though a single *Galiteuthis* species is recognized in Atlantic waters, molecular data suggest that at least 2–3 species are present, but whether they are new cryptic species or new geographic records of currently recognized species not present in the molecular databases is unclear. What is clear is that the species labeled here as *Ga. armata* actually represents that species, as this database includes a Mediterranean specimen (type locality, Sweeney 2017).

*Abraliopsis morisii* and *Abraliopsis* sp. 2 co-occur north of the Canary Current. The former is the only *Abraliopsis* species reported in the area. As the second was a paralarva, it is not possible to assess whether it is a new species or a new record for an already known species. *Mastigoteuthis agassizii* and *M*. cf. *dentata* were mainly recovered as the same species, but some analyses found cryptic biodiversity, so further studies using more markers are needed to assess whether cryptic species exist or whether the divergence simply reflects population structure. *Grimalditeuthis bonplandi* and *Py. margaritifera* did not show signals of cryptic biodiversity in Atlantic waters, but South Pacific *Py*. aff. *margaritifera* was identified as a different species by three of five species delimitation analyses. The six species currently recognized in the family Pyroteuthidae were present in the *cox1* database, which suggests that at least 7–10 species exist. If 12S *Pterygioteuthis* sp. 2 is confirmed as another cryptic species within *Pt. gemmata*, then there are 8–11 species within the family. Interestingly, *Pt. giardi* was present in two sister clades and one more distant lineage, suggesting convergent evolution of its morphology or misidentifications. Misidentifications are relatively common in the GenBank database (Groenenberg et al. 2009; Lindgren et al. 2012; Fernández-Álvarez et al. 2021; Lupše et al. 2023).

### Effects of oceanic currents over speciation in oceanic squids

Many cephalopod genera and cryptic species complexes show structured biogeographic patterns associated with past major biogeographic breaks, such as the Isthmus of Panama, oceanic features such as currents, or river discharges affecting salinity in large oceanic areas (e.g., Ibañez et al. 2016; Sales et al. 2017; Amor et al. 2019; Lima et al. 2020; Costa et al. 2021; Sanchez et al. 2021; Deville et al. in press). Four of the studied species (*C. sicula, C. canariensis, An. lesueurii*, and *Ga. armata*) showed strong signals of cryptic biodiversity. The species included in this work can be considered relatively passive against oceanic currents since they are either weakly muscled ammoniacal or small-bodied species. Thus, allopatric speciation triggered by reproductive isolation due to oceanographic features was expected. As Sayre et al. (2017) pointed out, ecological marine units have a greater geographical extent in deeper waters. Thus, a higher tendency for cryptic biodiversity among epipelagic species was expected. However, no connection between lifestyle and cryptic biodiversity was found among the 16 focal species (Table 1). Neon flying squid species boundaries are related to the main oceanographic currents (Fernández-Álvarez et al. 2020), and it had been hypothesized that these currents are too strong to allow connectivity among populations of neon flying squid populations, which have small paralarvae (Fernández-Álvarez et al. 2017, 2018), triggering speciation. Instead of being weakly developed detritivores, the paralarvae of the 16 focal species are thought to be predatory and more developed at hatching, and, in some cases, they can attain large sizes (e.g., *Le*. cf. *atlantica* and *Gr. bonplandi*), likely extending the paralarval dispersion period. Also, spawning in deep waters might promote dispersion across oceanic currents. While flying squids spawn in shallow waters (Staaf et al. 2008; Punneta et al. 2015), at least *Li. reindhartii* and *T. megalops* are believed to spawn in deep water (Sweeney et al. 1992). It is likely that the genus *Chtenopteryx* also spawns in deep waters, as does *Bathyteuthis* (Bush et al. 2012). Interestingly, the diamondback squid also reproduces in shallow waters and comprises allopatric cryptic species similar to the neon flying squid (Deville et al. in press). Although Atlantic oceanic currents can represent hard and soft barriers for plenty of species of reef fish (Floeter et al. 2008), only *Teuthowenia* spp. and the lineages of *H. navossae* spp. 1 and 2 and *Helicocranchia* sp. appeared affected by currents, specifically the Canary Current and the Atlantic Equatorial Current.

## Conclusions

Due to inconsistencies among the results of different markers and different species delimitation approaches, the scarcity of molecular data for oceanic squids distributed across large geographic areas, and the difficulties of finding morphological diagnostic characters for early life stages, it is difficult to assess the species boundaries for many of these species. Thus, we can say they are in the “grey speciation zone,” where the species taxonomy can be considered controversial (Roux et al. 2016). As many oceanic squids have cosmopolitan distributions, new studies combining genomic and morphological information from specimens collected worldwide are needed to correctly assess the actual oceanic squid biodiversity.

## Supplementary Material

icad116_Supplemental_Files

## Data Availability

The data underlying this article are available in the GenBank Nucleotide Database at https://www.ncbi.nlm.nih.gov/genbank/ and can be accessed with the GenBank accession numbers listed in Supplementary Table S1. The FastQ files can be accessed within the GenBank Nucleotide Database with the BioProject accession number PRJNA951074.
