# Supplementary material for: Atlantic Oceanic Squids in the “Grey Speciation Zone”
Source: Integr Comp Biol. 2023 Aug 21;63(6):1214–25. doi: 10.1093/icb/icad116 (PMC10755182; doi:10.1093/icb/icad116)
Supplement: icad116_Supplemental_Files [file icad116_supplemental_files.zip › icb-2023-0076-File005.pdf]

Integrative and comparative Biology

Atlantic oceanic squids in the "grey speciation zone"

Fernando Á. Fernández-Álvarez [1], Gustavo Sanchez [2], Diego Deville [3], Morag Taite [4], Roger Villanueva [1] & A. Louise Allcock [4]*

[1] Institut de Ciències del Mar (ICM), CSIC, Passeig Marítim de la Barceloneta 37-49, 08003 Barcelona, Spain; 0000-0002-8679-7377; f.a.fernandez.alvarez@gmail.com
[2] Molecular Genetics Unit, Okinawa Institute of Science and Technology, Onna, Okinawa 904-0412, Japan
[3] Graduate School of Integrated Sciences for Life, Hiroshima University, Higashihiroshima, Hiroshima 739-8528, Japan.; 0000-0002-9396-4671; diegodeville1608@gmail.com
[4] Ryan Institute and School of Natural Sciences, University of Galway, University Road, Galway H91 TK33, Ireland; 0000-0002-4806-0040; louise.allcock@universityofgalway.ie

*Correspondence: louise.allcock@universityofgalway.ie

Supplementary material

Contents

Supplementary material and methods **2**
Nomenclature *3*
PCR and Sanger sequencing *3*
Genome skimming *3*
Phylogenetic and molecular species delimitation analyses *4*
Supplementary results **6**
Species delimitation analyses *6*
Intra- and interlineage p*-distances* *16*
Species distribution ranges *26*
Supplementary references including those from Table S1 **36**

Table S1 can be accessed as a spreadsheet following the doi link of the article.

Supplementary material and methods

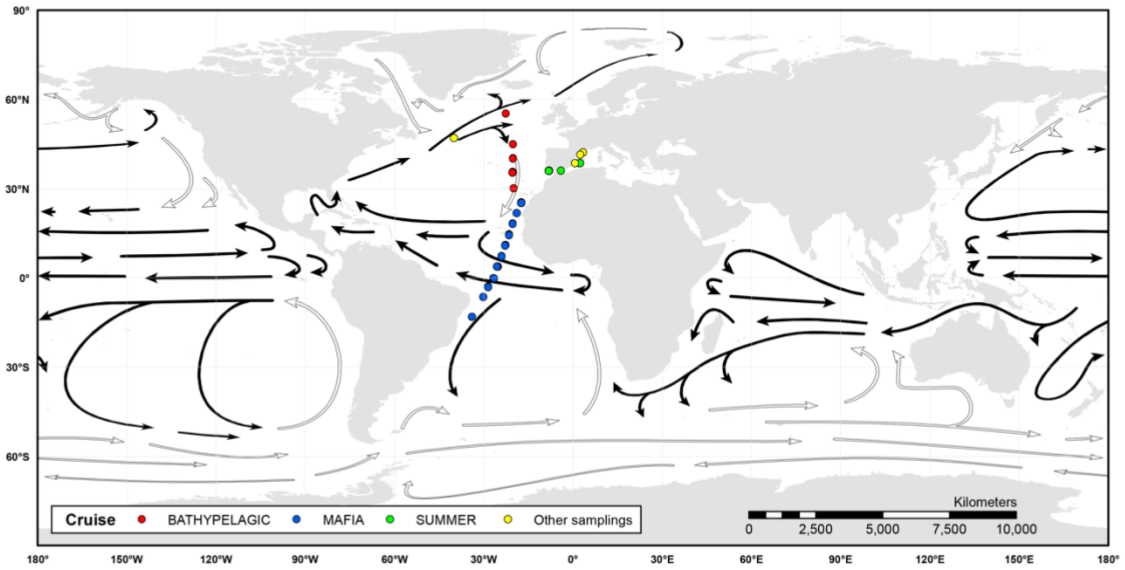

Fig. S1. Sampling localities of the newly sequenced specimens. For oceanic cruises, only sampling localities with squids studied here are shown. For more accurate information on sampling coordinates, see Table S1.

Nomenclature

In this work, the unified species concept (De Queiroz 2007) was applied. This concept treats the existence of separately evolving metapopulation lineages as the only necessary criterion to define species, regardless of which secondary criteria of lineage differentiation (e.g., intrinsic reproductive isolation, diagnosability or monophyly) have been met. We considered concordance of molecular species delimitation methods as evidence of the reproductive isolation between groups and therefore the species status for each group. Where there were inconsistencies among the results of different markers and different species delimitation approaches, and where morphological diagnostic characters were lacking, we considered lineages to be in the "grey speciation zone".

Nominal species distributed on both sides of one or more oceanic barriers were selected for this study, including individuals identified based on morphology as 16 different species: *Chtenopteryx sicula*, *Chtenopteryx canariensis*, *Ancistrocheirus lesueurii*, *Leachia atlantica*, *Liocranchia reinhardti*, *Bathothauma lyromma*, *Egea inermis*, *Teuthowenia megalops*, *Teuthowenia maculata*, *Helicocranchia navossae*, *Galiteuthis armata*, *Abraliopsis morisii*, *Grimalditeuthis bonplandi*, *Mastigoteuthis agassizii*, *Pyroteuthis margaritifera* and *Pterygioteuthis gemmata*. We completed each gene matrix by adding sequences of the same genus from GenBank. For convenience, divergent clades among these morphospecies received different names (see Table S1). When a sequence from a location near the type locality of the species was present in a particular clade, that clade received the name of the nominal species, and subsequent divergent clades morphologically identified as the same morphospecies received other names. These names were case-sensitive and, when possible, the original denomination provided by previous GenBank submitters was used (the primary literature of these sequences is summarized in Table S1).

Chtenopteryx cf. *canariensis* was named that way as it did lack ocular and visceral photophores as typical for *C. canariensis*, but molecular data suggest it is not the nominal species and also we are not sure in which ontogenetic stages these photophores appear. *Chtenopteryx*. cf. *sicula* spp. 1–4 were identified as *C. sicula* in their original submissions, but molecular and distribution data suggest they are not the nominal species. *Chtenopteryx* spp. KER1, KER2 and C retain the original name from their authors. *Ancistrocheirus* spp. 1-3 are named as such as the type locality of the nominal species of this monotypic genus was not covered by the current sampling and, therefore, we cannot allocate any of the identified clades as that species. *Leachia* cf. *atlantica* was identified as such as the sampling was based on immature specimens and the 18S *Le. atlantica* sequence AY557488 was the sister group of *Le.* cf. *atlantica* with *Leachia lemur*. As there is no *cox1*, 12S or mitoPCG data from the specimen that yielded AY557488 that can be used to see if this paraphyletic assemblage is due to incomplete lineage sorting or otherwise, we decided to keep both *Le. atlantica* 18S clades as separate units. *Helicocranchia navossae* spp. 1 and 2 were treated as different clades as preliminary results pointed towards possible cryptic biodiversity. *Helicocranchia pffefferi* spp. 1–3 are divergent clades that were identified as *H. pffefferi*, but we are unsure which, if any, represents the nominal species. *Helicocranchia* sp. and *Helicocranchia* sp. KER retained their original names. As *Ga. armata* specimens from near the type locality were present, one of the clades received the nominal species name and the other clades formed by individuals identified as *Ga. armata,* or not identified at the species level, were named *Galiteuthis* spp. 2 and 3. *Galiteuthis* sp. KER retained the name assigned by the submitting authors. *Abraliopsis* sp. 1 was identified from morphology as *Ab. morisii*, but another divergent clade including individual of this species from near the type locality already existed. *Abraliopsis* sp. 2 was a paralarva without diagnostic characters. *Abraliopsis* sp. and *Abraliopsis* sp. 3 were not identified beyond genus level by the submitting authors. *Mastigoteuthis* cf. *dentata*, *Py.* aff. *margaritifera* and *Pt.* cf. *gemmata* were identified as such in the original GenBank submissions. *Pterygioteuthis* sp. 2 are individuals identified from morphology as *Pt. gemmata*, but their 12S sequences are divergent. Sequences identified as *Pt. giardi* are present in three clades, two of which also include sequences identified as *Pyroteuthis* RJ-2009 and *Pterygioteuthis hoylei*. As we were uncertain which of these clades actually represents *Pt. giardi*, the name was used for all three. In total, 56 clades were named (see Table S1 for more details).

PCR and Sanger sequencing

DNA barcoding, which targets a fragment of the *cytochrome c oxidase* (*cox1*) gene, was performed using forward primer LCO1490 and reverse primer HC02198 (Folmer et al. 1994), and a small fragment of 12S rRNA using the new primers IKAF (5'–AAG TGG TTA AAT TGG TGC CAG C–3') and IKAR (5'–AAG AAT AAT AGG GTC TCT AAT CCT AG–3'), on 127 samples and 131 samples, respectively (Table S1). Each PCR contained 6.25 μL of Thermo Scientific™ DreamTaq Green PCR Master Mix (2X), 0.5 μM of each primer, 1 μL of DNA and 4.8 μL H_20. A PCR negative control was also included in each round to detect possible contaminations. The PCR conditions were 94 °C for 2 min, 35 cycles of 94 °C for 15 s, annealing temperature for 30 s and 72 °C for 90 s, followed by 72 °C for 10 min. The annealing temperature was 50 ºC for *cox1* and 45 ºC for 12S. PCR products were visualized on a 1% (w/v) agarose gel stained with SYBR Safe (Thermo Fisher Scientific, MA, US). PCR products were cleaned using an Invitrogen™ PureLink™ PCR Purification Kit according to the manufacturer's instructions. Samples were sent to Eurofins Genomics (Germany) for DNA sequencing

on an ABI 3730XL DNA Analyzer. All electropherograms were checked using 4 Peaks (Nucleobytes™) and aligned in UGENE (Okonechnikov et al. 2012)

Genome skimming

Shallow whole genome sequencing data were produced through genome skimming (Dodsworth 2015), which allows the recovery of large and high copy loci of the genome, such as the complete mitogenome and the nuclear 18S ribosomal subunit. Indexed libraries were prepared using a BGI Library Kit and sequenced 9 Gb/sample in an DNBseq-G400 (Beijing Genomics Institute, Shenzhen, China). The quality of the reads was assessed through FastQC (Andrews 2010). Mitochondrial and nuclear ribosomal DNA were assembled de novo using NOVOPlasty3.8.3 (Dierckxsens et al. 2016) using a reference sequence (either the complete mitogenome or the complete nuclear ribosomal gene cluster of a closely related species), and a fragment of *cox1*, 12S or 16S rRNA (for the mitogenomes) or a fragment of 18S or 28S rRNA (for 18S rRNA) as a seed. For mitogenome gene annotations Mitos2 (Bernt et al. 2013) was used, with NCBI Ref-Seq 63 Metazoa database reference and genetic code 5, for invertebrates. Gene annotations were checked and corrected by hand. NOVOPlasty uses a short sequence as a seed and extends it based on overlapping reads. This is problematic in oceanic squids with duplicate genes and causes the program to output different sets of contigs, each containing one copy of the duplicate gene (see Fernández-Álvarez et al. 2022). For solving this methodological artifact, we used the mitogenome gene orders established for the flying squids *Todarodes pacificus* and *Watasenia scintillans* by Yokobori et al. (2004) using long PCRs. Nuclear 18S genes were annotated using RNAmmer (Lagesen et al. 2007).

Phylogenetic and molecular species delimitation analyses

Maximum Likelihood analyses (ML) were performed on the IQTREE server (Hoang et al. 2018, Nguyen et al. 2015). The statistical support for each node was indicated after 2,000 ultrafast bootstrap iterations. For selecting a model, we implemented the ModelFinder tool (Kalyaanamoorthy et al. 2017) in the IQTREE portal to estimate the best fitting model of substitution for each partition following the Bayesian Information Criterion (BIC), and selected the appropriate model for each database for the downstream analyses. A coalescent phylogenetic inference analysis was performed in BEAST v. 2.7.3 (Bouckaert et al. 2019). The input file was created using BEAUti. Site and clock models were set based on the results of ModelFinder and selected using the extended options of the BEAST Package Standard Substitution Models SSM v. 1.2.0 (Bouckaert and Xie 2017). Clock models were set to relaxed log-normal models (Drummond et al. 2006). The prior of the species tree model was set to Yule model, and the birth rate was estimated by the analysis. *Spirula spirula* was added to the matrix as an outgroup in the time-calibrated Bayesian Inference (BI) analysis. The order Oegopsida, the genus *Chtenopteryx,* and oceanic squids (i.e., Oegopsida + Bathyteuthida), were set up as monophyletic based on the results of Strugnell et al. (2017). Additionally, a fossil calibration was applied to the analyses: *Spirula spirula* and descendants with a minimum age of 75 Ma (see Tanner et al., 2017). For each analysis, a Markov Chain (Drummond et al. 2002) of 100 million generations was run sampling every 10,000 generations. Chain convergence was examined with Tracer v. 1.7.2 (Rambaut et al. 2018) and ESS values were checked to ensure they were over 200. Finally, the initial 25% tree configuration was discarded as burn-in and the majority consensus tree obtained using TreeAnnotator.

We ran five different species delimitation methods. For running the Bayesian Poisson Tree Processes (bPTP), the obtained ML tree was visualized and converted to Newick format using FigTree v.1.4.3 (Rambaut 2010) and submitted to the bPTP portal (http://species.h-its.org/ptp/) using the default parameters. Based on FAF-A's previous experiences with this analysis, only the ML solution of the analysis was considered, as the Bayesian solution commonly produces unreliable results with cephalopods. The ASAP method (Puillandre et al. 2020) was performed using p-distances in the webserver (https://bioinfo.mnhn.fr/abi/public/asap/) and only the option with highest likelihood score was considered. TCS v.1.21 (Clement et al. 2000) was used to construct the haplotype networks with a maximal connectivity limit of 95%, which empirically commonly reflect the species assemblages (e.g., Kang et al. 2015). For the ribosomal genes, INDELs were codified as missing characters to avoid over-splitting the haplotype networks. For running the Generalized Mixed Yule Coalescent approach (GMYC, Fujisawa and Barraclough, 2013), the Maximum Credibility Tree of the BI analysis was transformed into Newick format and submitted to the GMYC portal (http://species.h-its.org/gmyc/). Both the single and multi threshold approaches of the GMYC were considered in this study. Additionally, we used a Bayesian implementation of GMYC in the R package bGMYC (Reid and Carstens 2012). As input, we used a set of 100 ultrametric trees generated from the output of the four BEAST2 analyses using LogCombiner. bGMYC uses the variation among the 100 trees to produce probability values for each species and a heatmap for easy visualization.

Supplementary results

Species delimitation analyses

The *cox1* database included 355 sequences belonging to 41 species (27 nominal and 14 unidentified species) and was 608 nucleotides (nt) long. The 12S matrix included 131 sequences belonging to the 16 focal species and was 212 nt long. The mitoPCG database included 25 sequences belonging to 15 species, and was 11,208 nt long. Finally, 18S included 39 sequences belonging to 21 nominal species and was 2,850 nt long. The BIC criterion selected the models TIM2+F+I+G4 for *cox1*, HKY+F+G4 for 12S, and GTR+F+I+G4 for mitoPCG and 18S.

The *cox1* database was the largest among our datasets with 355 sequences and 47–56 species belonging to 14 genera, with a large representation of both Atlantic and Pacific sequences. The bPTP, ASAP, TCS, sGMYC and mGMYC analyses recognized 56, 47, 50, 43 and 48 species, respectively (Fig. 1) North Atlantic, Mediterranean and North Pacific individuals of *C. sicula* were split into three to four species, as the North Pacific *C.* cf. *sicula* spp. 2 and 3 were either recovered as a different species or the same. *Chtenopteryx* cf. *canariensis* and *C. canariensis* were recovered as different species by all analyses except mGMYC. The South Atlantic *C.* cf. *canariensis* was recovered conspecific with South Pacific *Chtenopteryx* sp. KER 1 in the sGMYC analysis. In addition to these two nominal species, there are at least one to three *Chtenopteryx* spp. (KER 1, KER 2 and C) depending on different species delimitation analyses. Excluding bPTP, all analyses recognized three *Ancistrocheirus* species, two occurring in North Atlantic waters, and another in the North Pacific. The three *Leachia* species were recognized as different species in all analyses without signals of additional cryptic biodiversity for this marker. *Liocranchi reinhardtii* and *Li. valdiviae*, *B. lyromma* and *E. inermis* were each recovered as a single species in all analyses except mGMYC which split *Li. reinhardtii* in four. Species delimitation of *Teuthowenia* spp. varied greatly by analysis: while *T. pellucida* was isolated from the other two *Teuthowenia* species in all analyses, *T. megalops* and *T. maculata* were recovered as conspecific by both GMYC analyses. *Teuthowenia megalops* was split in two by the bPTP analysis. All *H. navossae* were recovered as conspecific by all analyses except GMYC, and the ASAP and sGMYC analyses recovered them as conspecific with the South Atlantic *Helicocranchia* sp. sequence KF369197. North Atlantic and Mediterranean *Ga. armata* sequences were recovered as a single species isolated from other *Galiteuthis* spp. sequences. North Atlantic *Galiteuthis* sp. 2 were recovered as conspecific with the South Pacific sequence *Galiteuthis* sp. KER by the bPTP and TCS analyses, and as different species by the remaining analyses. South and North Atlantic *Galiteuthis* sp. 3 were recovered as different species from the previously mentioned *Galiteuthis* clades in all analyses. Three clades assigned to *Ab. morisii* in this work (*Ab. morisii* and *Abraliopsis* spp. 1 and 2) were classified as different species by all species delimitation analysis, but both versions of GMYC recovered *Abraliopsis* sp. 1 as conspecific with *Ab. atlantica*. *Grimalditeuthis bonplandi* was recovered as a single species by all analyses except bPTP which split it in two. Sequences labeled as *M. agassizii* and *M.* cf. *dentata* from the North and South Atlantic and South Pacific were present in GenBank. They were recovered as a single species by the bPTP, ASAP and TCS analyses, but both GMYC analyses recovered them as three species: one included North and South Atlantic *M. agassizii* specimens, another that included North Atlantic and South Pacific *M. agassizii* sequences, and finally one that included all specimens labeled as *M.* cf. *dentata*. The sister groups North Atlantic *Py.*

margaritifera and South Pacific *Py.* aff. *margaritifera* were recognized either as different species (bPTP, ASAP, sGMYC) or conspecific (TCS, mGMYC). While North Atlantic *Pt. gemmata* and South Pacific *Pt.* cf. *gemmata* were unambiguously recognised as different species by all analyses, *Pt.* cf. *gemmata* was recovered as conspecific with the South Atlantic *Pt. giardi* sequence GU145065 in both *cox1* GMYC analyses. Time calibrations of the coalescent analysis are available in Fig. S1.

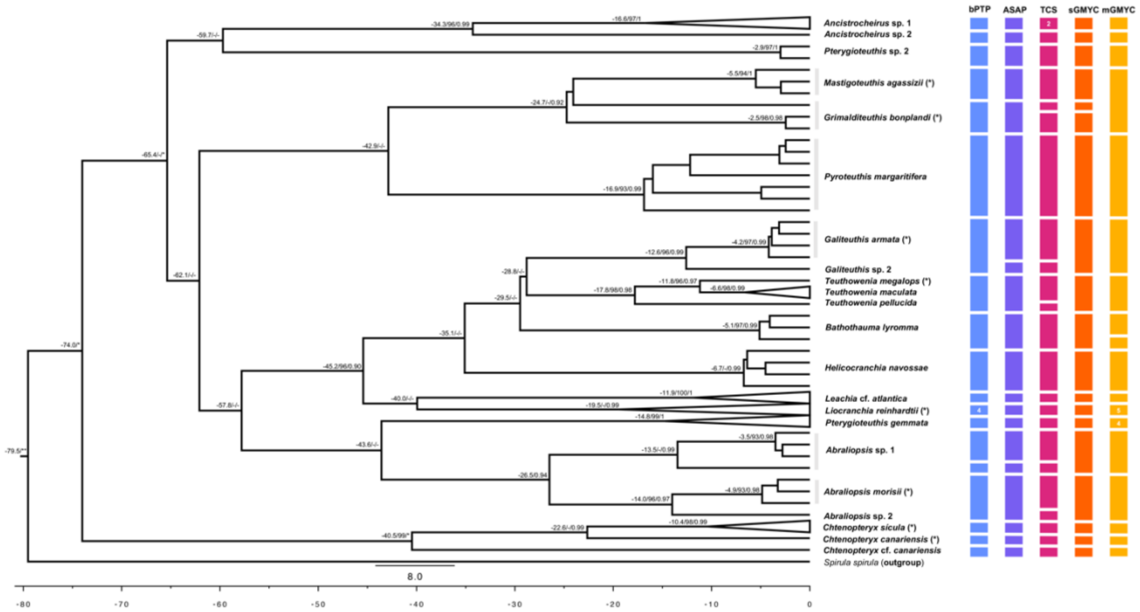

Fig. S2. Summary of species delimitation analyses obtained with the 12S database, depicted over the coalescent analyses obtained through BEAST 2.6.4 (Bouckaert et al. 2019). For convenience, clades formed by six or more sequences were collapsed. Numbers above branches designate the node ages (Mya), the ultrafast bootstrap percentages (%) from the ML analysis, and the posterior probabilities from the coalescent analysis. Bootstrap and posterior probabilities of less than 90 % and 0.9 respectively were not recorded. Dashes indicate lack of support or clades with a different topology in the ML analysis. Asterisks designate clades that were constrained as monophyletic for the coalescent analysis, double asterisks designate the time calibration. Asterisks inside parenthesis designate lineages with one or more sequences coming from a locality close to the type location of a nominal species. Vertical lines summarize the results from bPTP, ASAP, TCS, sGMYC and mGMYC (see supplementary material and methods for more details). Numbers inside some results for delimitation analysis summarize the number of species found for a collapsed clade.

The 12S database included 131 sequences, 16 nominal species and 19–25 nominal species which belong to 13 genera. The bPTP, ASAP, TCS, sGMYC and mGMYC recognized 22, 20, 24, 19 and 25 species. Several species present in the *cox1* database were not present. All species delimitation analyses from the 12S database showed differences among them (Fig. S2). *Chtenopteryx sicula*, *C. canariensis*, *C.* cf. *canariensis*, *Le.* cf. *atlantica*, *H. novossae* and *Py. margaritifera* were unambiguously recovered as different species by the five species delimitation analyses. *Ancistrocheirus* spp. 1 and 2 were unambiguously recovered as different species in every analysis. *Ancistrocheirus* sp. 1 was recovered as a single species in all analyses except TCS, where it was split in two. *Liocranchia reinhardtii* was recovered as a single species in all analyses but bPTP and mGMYC. *Bathothauma lyromma* was assigned to a single species in all analyses except mGMYC, where one of the three sequences were recognized as a separate species. The

three *Teuthowenia* species were recovered as a single species by all species delimitation analyses except TCS, in which *T. pellucida* was recovered separately, and *T. megalops* and *T. maculata* were recovered together as a second species. *Galiteuthis armata* and *Galiteuthis* sp. 2 were recovered together as a single species in three analyses (bPTP and both GMYC). *Abraliopsis morisii* and *Abraliopsis* sp. 2 were recovered together as a single species in all analyses except TCS. A divergent haplotype of *Abraliopsis* sp. 1 was recovered as a different species by the bPTP, ASAP and TCS analyses. *Grimalditeuthis bonplandi* was recovered as a single species by bPTP and ASAP, however TCS and sGMYC split the North Atlantic sample from the two South Atlantic sequences. mGMYC recovered *Gr. bonplandi* and *M. agassizii* as a single species. However, it must be noted that in the BI analysis *Gr. bonplandi* is paraphyletic including *M. agassizii*, likely creating artifacts in both GMYC versions. Two individuals originally identified as *Pt. gemmata* (*Pterygioteuthis* sp. 2), but for which *cox1* sequencing failed, were recovered separately to *Pt. gemmata*. In the absence of *cox1* it is difficult to assess whether these samples actually represent a different species or if they simply have divergent 12S haplotypes. The remaining *Pt. gemmata* samples were recovered as a single species, but mGMYC split this clade into four different species.

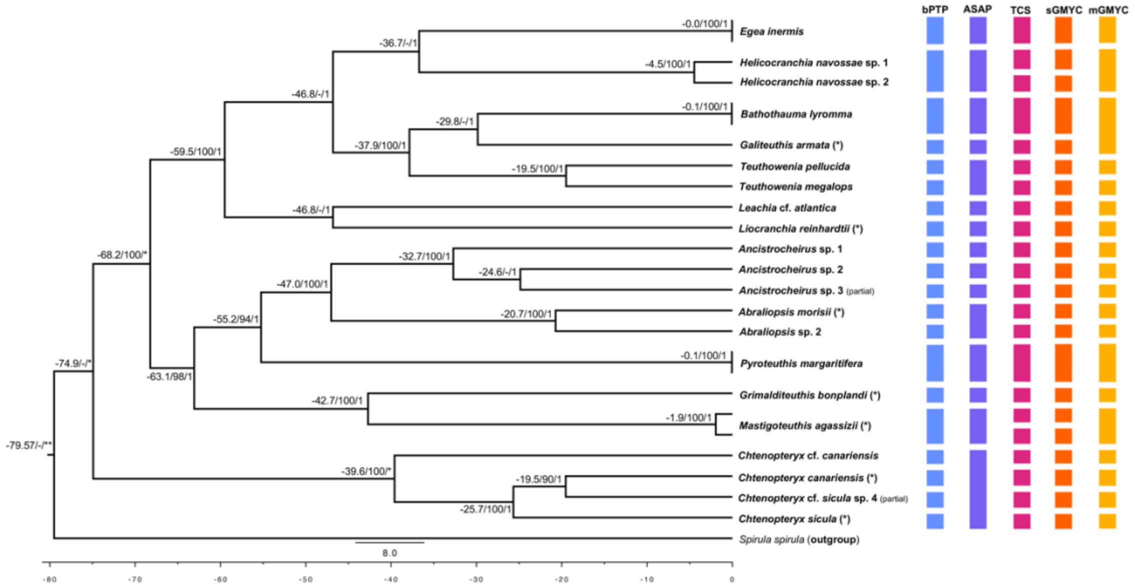

Fig. S3. Summary of species delimitation analyses obtained with the mitoPCG database, depicted over the coalescent analyses obtained through BEAST 2.6.4 (Bouckaert et al. 2019). Numbers above branches designate the node ages (Mya), the ultrafast bootstrap percentages (%) from the ML analysis, and the posterior probabilities from the coalescent analysis. Bootstrap probabilities of less than 90 % were not recorded. Dashes indicate lack of support or clades with a different topology in the ML analysis. Asterisks designate clades that were constrained as monophyletic for the coalescent analysis, double asterisk designate the time calibration. Asterisks inside parenthesis designate lineages with one or more sequences coming from a locality close to the type location of a nominal species. Vertical lines summarized the results from bPTP, ASAP, TCS, sGMYC and mGMYC (see supplementary material and methods for more details).

With 11,208 nt, mitoPCG was the longest database but with the lowest number of analyzed sequences (25, Fig. S3). It included 15 nominal species, which belong to 13 genera. bPTP, ASAP, TCS, sGMYC and mGMYC recognized 20, 15, 22, 22 and 19 different species, respectively. *Teuthowenia maculata*, *Galiteuthis* sp. 2, *Abraliopsis* sp.

1 and *Pt. gemmata*, which are present in both *cox1* and 12S matrices, were not present due to failures of the BGI Library Kit prep. For the same reason, there are no intraspecific mitogenomes of *Le.* cf. *atlantica*, *Li. reinhardtii* or *Gr. bonplandi*. The partial mitogenomes of *Chtenopteryx* cf. *sicula* sp. 4 and *Ancistrocheirus* sp. 3 were mined from GenBank. *Chtenopteryx* spp. were each recovered as different species except in the ASAP analysis, where all were recovered as members of a single species. *Ancistrocheirus* spp. and most singleton genera (*Le. cf. atlantica*, *Li. reinhardtii* and *Gr. bonplandi*) were each recovered as a unique species in all delimitation analyses. *Bathothauma lyromma* and *Galiteuthis armata* were recovered united as one species by mGMYC. *Egea inermis* was recovered as a single species in all analyses. *Teuthowenia megalops* and *T. pellucida* were recovered as unique species in all analyses except ASAP, in which they were united as a single species. *Helicocranchia novossae* and M. agassizii sequences were recovered as a single species each except in the TCS and the sGMYC. *Abraliopsis morisii* and *Abraliopsis* sp. 2 were recovered as different species but in the ASAP analysis. The two *Py. margaritifera* sequences were recovered as a single species by all analyses.

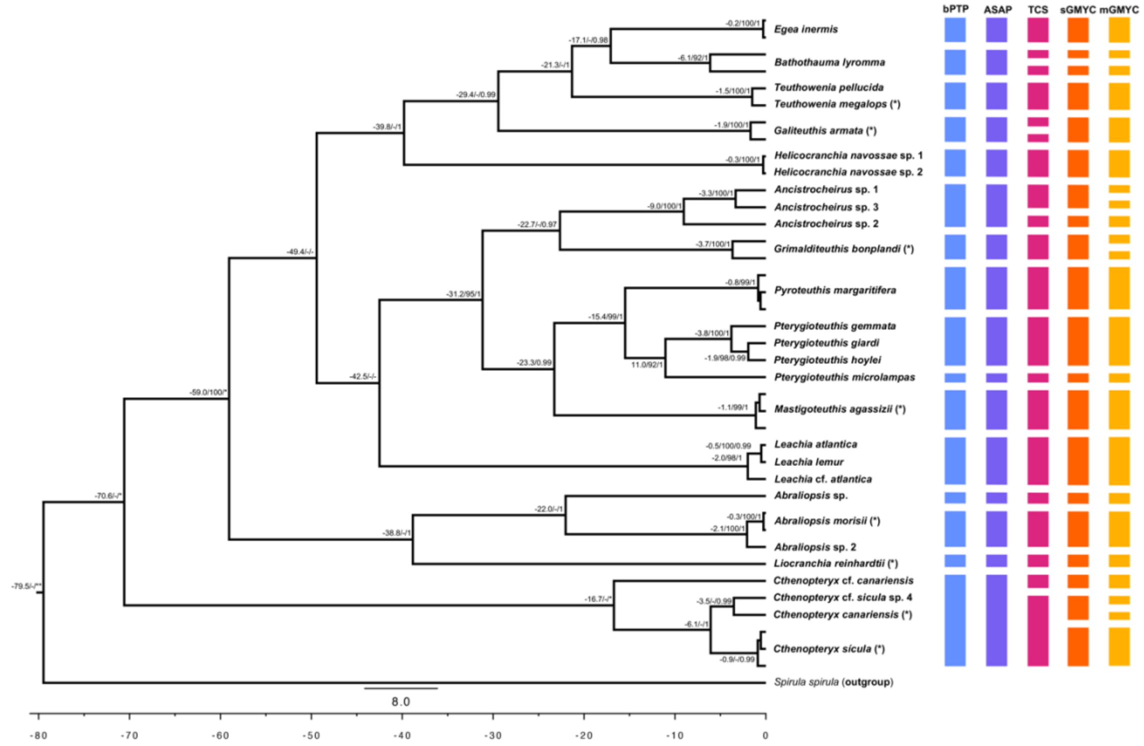

Fig. S4. Summary of species delimitation analyses obtained with the 18S database, depicted over the coalescent analyses obtained through BEAST 2.6.4 (Bouckaert et al. 2019). Numbers above branches designate the node ages (Mya), the ultrafast bootstrap percentages (%) from the ML analysis, and the posterior probabilities from the coalescent analysis. Bootstrap and posterior probabilities of less than 90 % and 0.9 respectively were not recorded. Dashes indicate lack of support or clades with a different topology in the ML analysis. Asterisks designate clades that were constrained as monophyletic for the coalescent analysis, double asterisk designates the time calibration. Asterisks inside parenthesis designate lineages with one or more sequences coming from a locality close to the type location of a nominal species. Vertical lines summarized the results from bPTP, ASAP, TCS, sGMYC and mGMYC (see supplementary material and methods for more details).

The 18S matrix was represented by 39 sequences labeled as 21 nominal species belonging to 14 genera. bPTP, ASAP, TCS, sGMYC and mGMYC recognized 16, 16, 20, 20 and 23 nominal species. The 18S matrix lacks the same samples as the mitoPCG matrix due to failures in BGI Library Kit prep. The matrix was enriched by sequences of *Ga. armata*, *Ab. morisii*, *Gr. bonplandi*, *M. agassizii*, *Py. margaritifera*, *Pt. gemmata* and additional species of the genera *Chtenopteryx*, *Ancistrocheirus*, *Leachia*, *Abraliopsis* and *Pterygioteuthis* mined from GenBank. In general, species delimitation analyses based on the 18S database recognized a lower number of species than the other databases (Fig. S4). bPTP and ASAP results were identical and recognized 16 species, a lower number than other species delimitation methods. All *Chtenopteryx* sequences were recognized as a single species in the bPTP and ASAP analyses, while TCS and both GMYC analyses recognized *C.* cf. *canariensis* as a separate species from the rest, sGMYC analysis recognized *C. sicula* and an additional species formed by *C. canariensis* and *C.* cf. *sicula* sp. 4, and mGMYC recognised all four named groups. All *E. inermis*, *H. novossae*, *M. agassizii* and *Py. margaritifera* were each unambiguously recorded as a single species by all species delimitation analyses. *Ancistrocheirus* spp. were recognized as a single species in bPTP and ASAP analyses, while *Ancistrocheirus* sp. 2 was recognized as separate by sGMYC, and the three sequences were each recognised as different species by mGMYC. Each of the congeneric pairs *Le. atlantica* and *Le. lemur* and *T. megalops* and *T. pellucida* were united as a single species per genus in all species delimitation analyses. The singletons *Li. reinhardtii* and *Abraliopsis* sp. were each considered as unique species in all analyses. The two sequences of *B. lyromma* were regarded as different species by TCS and both versions of the GMYC but united as a single species by the other delimitation methods. *Galiteuthis armata* was recognized as a single species by all methods except TCS. *Abraliopsis morisii* and *Abraliopsis* sp. 2 were recovered as a single species by all species delimitation analyses. *Grimalditeuthis bonplandi* was split into two species only by mGMYC. The congeneric *Pt. gemmata*, *Pt. giardi* and *Pt. hoylei* were united as a single species and recovered distinct from *Pt. microlampas* by all methods.

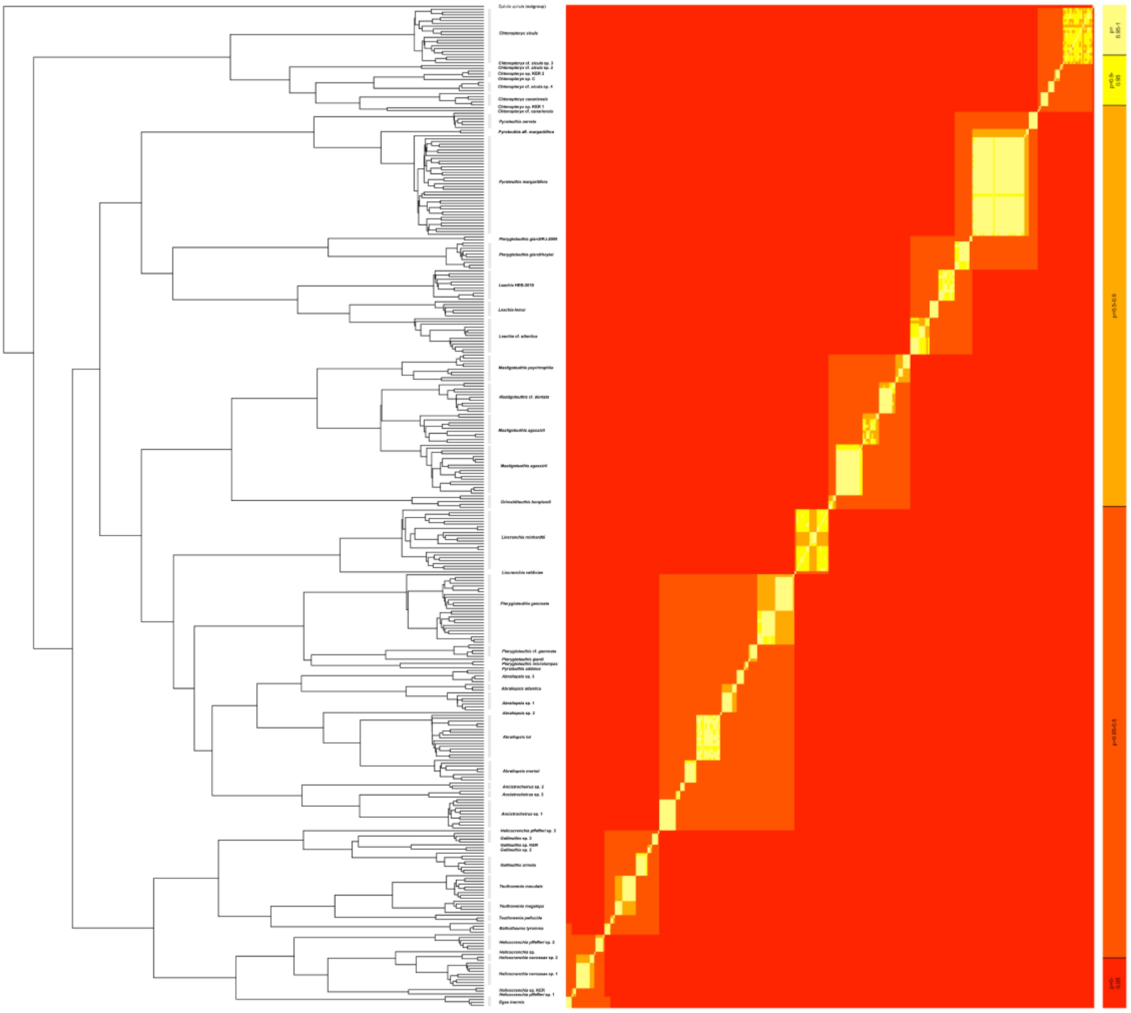

Fig. S5. bGMYC analysis of the *cox1* matrix depicted over the coalescent analyses obtained through BEAST 2.6.4 (Bouckaert et al. 2019). Heatmap color scale represents probability values according to the vertical scale on the right.

Results of bGMYC for *cox1* (Fig. S5) provided probability values of 0.9–0.95 or 0.95–1 for each of the nine taxonomic labels in which the genus *Chtenopteryx* is divided in this work (see Table S1). The probability of *Ancistrocheirus* spp. 1-3 each representing a different species is greater than 0.9. The probability of *Le.* cf. *atlantica*, *Li. reinhardtii*, *Abraliopsis* sp. 1 and *Pt. gemmata* of being a single species each was higher than 0.5. *Bathothauma lyromma* and *E. inermis* have a probability of being a single species each of 0.95–1. *Heliconcranchia navossae* spp. 1 and 2 have a probability of 0.5–0.9 of being the same species. The probability of this clade being the same species as *Helicocranchia* sp. is 0.05–0.5. *Teuthowenia megalops* and *T. maculata* have a probability of being a different species each of 0.95-1, while the probability of both being conspecific is 0.5–0.9. All *Ga. armata* have a probability of being the same species of 0.9–0.95. Representatives of *Galiteuthis* sp. 2 have a probability of 0.95–1 of representing a single species, and a probability of 0.5–0.9 of being conspecific with *Galiteuthis* sp. KER. The probability of *A. morisii* and *Abraliopsis* sp. 2 each being a single species was higher than 0.9. Two and three of the *Gr. bonplandi* sequences were split into two species with a probability higher than 0.9. The probability of all *Mastigoteuthis* sequences belonging to the same species was 0.05–0.5, and the probability of each of the *M. agassizii* and *M.* cf. *dentata* clades ranged from 0.5 to 0.95. The probability of *P. margaritifera* and *P.* cf.

margaritifera each being a different species is 0.9–0.95, while the probability of both of them being a single one is 0.05–0.5.

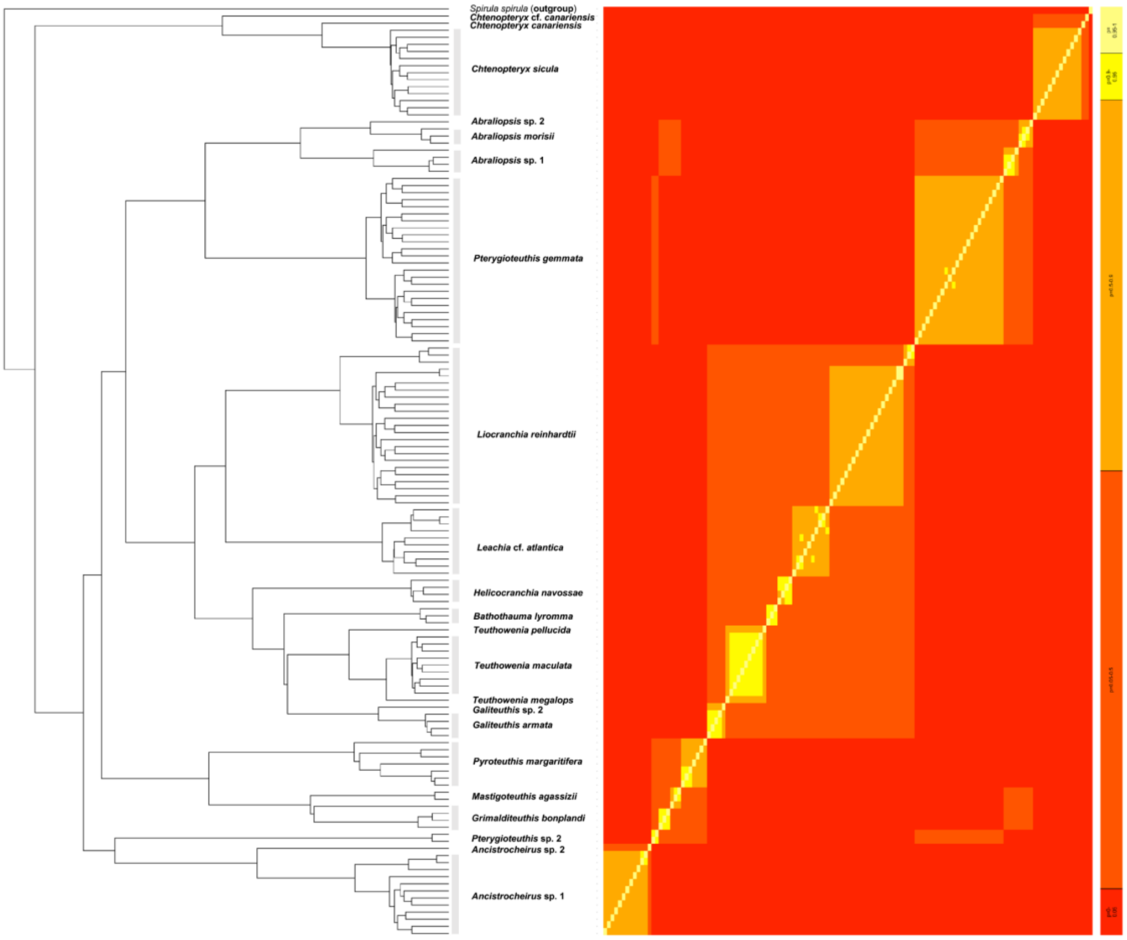

Fig. S6. bGMYC analysis of the 12S matrix depicted over the coalescent analyses obtained through BEAST 2.6.4 (Bouckaert et al. 2019). Heatmap color scale represents probability values according to the vertical scale on the right.

bGMYC on the 12S database (Fig. S6) yielded probability values of 0.05–0.5 for all three *Chtenopteryx* species present in this work being conspecific. A probability 0.95-1 was reported for *C. canariensis* and *C.* cf. *canariensis* being a single species each. The probability of all *C. sicula* sequences being conspecific is 0.5-0.9. *Ancistrocheirus* sp. 1 had a probability of conspecificity of 0.5-0.9, while the probability of *Ancistrocheirus* sp. 2 was 0.9–0.95. The probability that the two species of *Ancistrocheirus* are the same is 0.05–0.5. *Liocranchia reindhardtii* was split into two clades with a probability of 0.5–0.9. All sequences of *Le.* cf. *atlantica* were recognized as a single species with a probability of 0.5–0.9. The probability of conspecificity of *B. lyromma* was 0.9–0.95. The probability that all *H. navossae* sequences are conspecific is 0.5–0.95. The probability of the three nominal *Teuthowenia* species each representing a species was 0.9–0.95. The probability of all members of the genus being conspecific was 0.5–0.9. *Galiteuthis armata* and *Galiteuthis* sp. 2 had a probability of 0.9–0.95 of each being a species. The probability that the two are conspecific is 0.5–0.9. The probability of each of the two clades of *Grimalditeuthis bonplandi* being a single species was 0.9–0.95. There is a 0.5–0.9 probability *Ab. morisii* and *Abraliopsis* sp. 2 are the same species, the same as the probability of all sequences of *Abraliopsis* sp. 1 being conspecific. The probability that

M. agassizii is a single species was 0.5–0.9. The probability that *Pt. gemmata* and *Py. margaritifera* are each a single species was 0.5–0.9. The probability that *Pterygioteuthis* sp. 2 is a single species was 0.9–0.95.

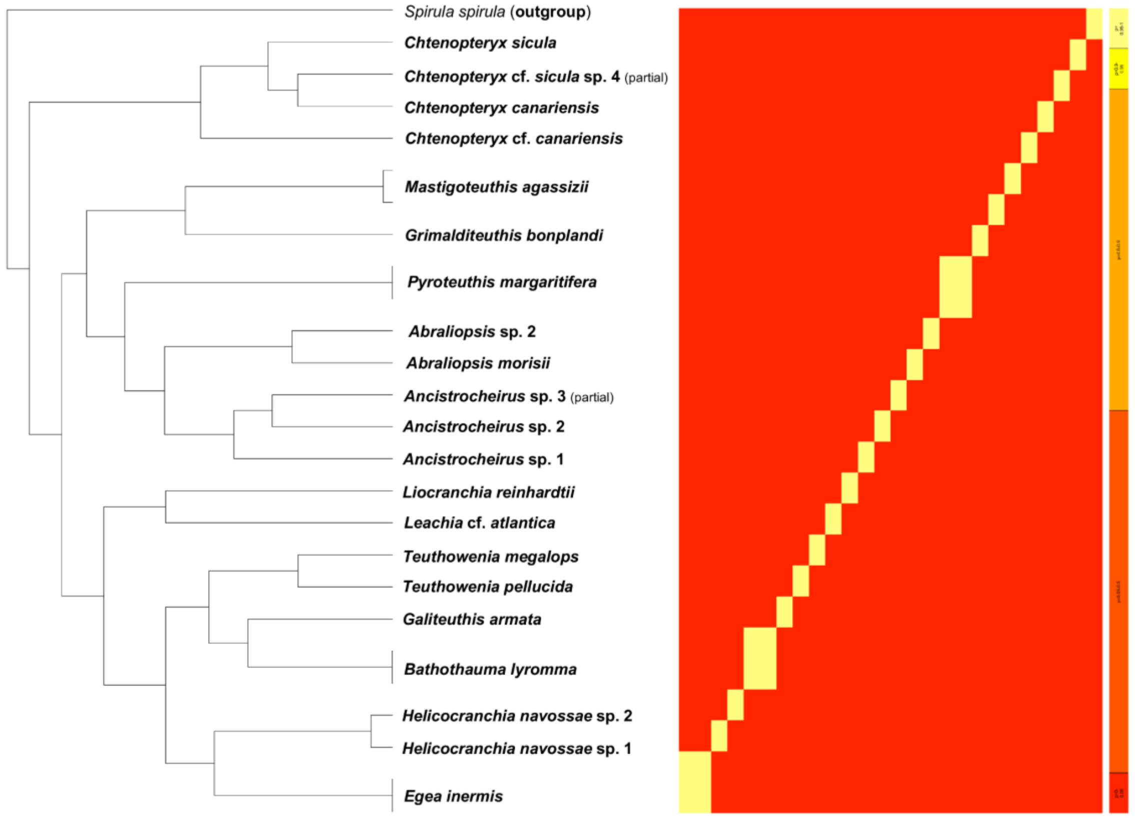

Fig. S7. bGMYC analysis of the mitoPCG matrix depicted over the coalescent analyses obtained through BEAST 2.6.4 (Bouckaert et al. 2019). Heatmap color scale represents probability values according to the vertical scale on the right.

Results of bGMYC for mitoPCG (Fig. S7) provided probability values of 0.95–1 for all the recognized species, and values 0-0.05 for all the remaining possibilities. The list of recognized species is *C. sicula*, *C.* cf. *sicula* sp. 4, *C. canariensis*, *C.* cf. *canariensis*, *Ancistrocheirus* sp. 1, *Ancistrocheirus* sp. 2, *Ancistrocheirus* sp. 3, *Li. reinhardtii*, *Le.* cf. *atlantica*, *E. inermis*, *B. lyromma*, *H. navossae* sp. 1, *H. navossae* sp. 2, *Ga. armata*, *T. megalops*, *T. pellucida*, *Gr. bonplandi*, *Ab. morisii*, *Abraliopsis* sp. 2 and *Py. margaritifera*. *Mastigoteuthis agassizii* was recognized as two species.

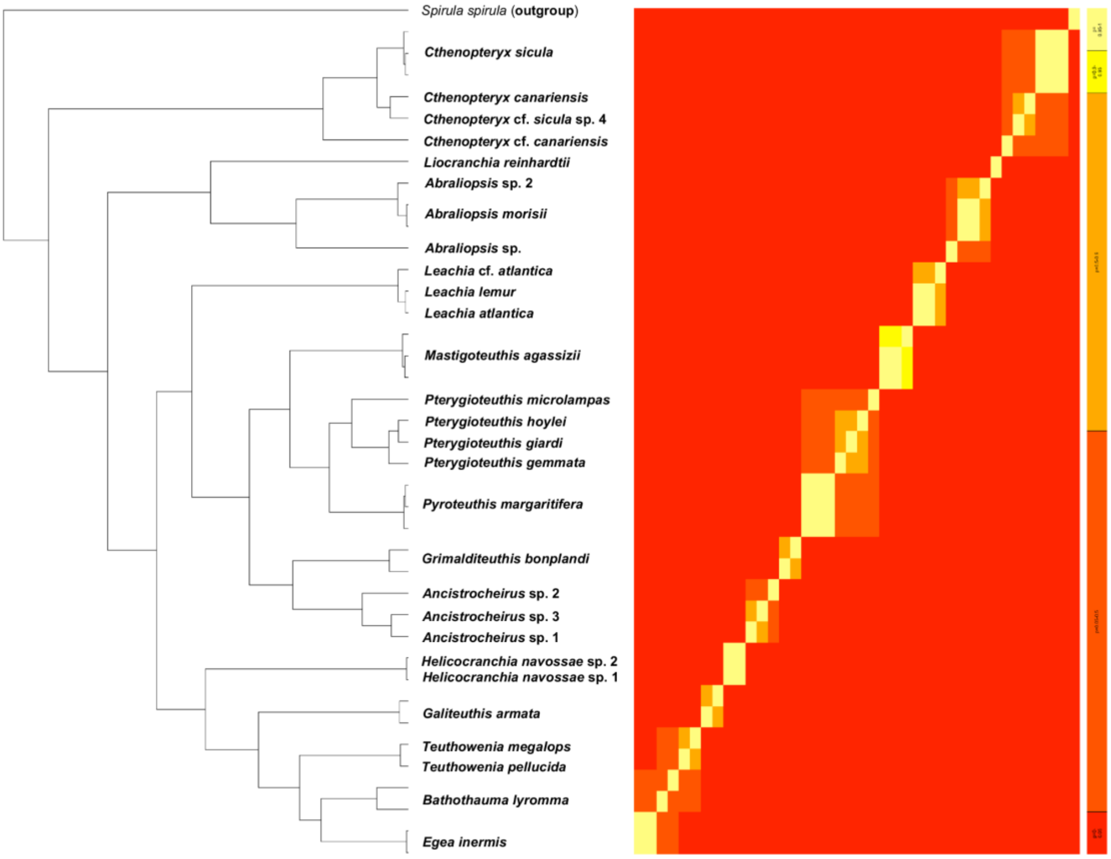

Fig. S8. bGMYC analysis of the 18S matrix depicted over the coalescent analyses obtained through BEAST 2.6.4 (Bouckaert et al. 2019). Heatmap color scale represents probability values according to the vertical scale on the right.

Results of bGMYC for mitoPCG (Fig. S8) recognized *C. sicula*, *C.* cf. *sicula* sp. 4, *C. canariensis* and *C.* cf. *canariensis* each as a single species with probabilities of 0.95–1. The probability of conspecificity of *C. canariensis* and *C.* cf. *sicula* sp. 4 was 0.5–0.9, and the probability of all species of the genus *Chtenopteryx* being conspecific was 0.05–0.5. *Ancistrocheirus* sp. 1, *Ancistrocheirus* sp. 2 and *Ancistrocheirus* sp. 3 were each recognized as a single species with probabilities of 0.95–1. The probability that *Ancistrocheirus* spp. 1 and 3 were conspecific was 0.5–0.9, and the probability of the whole genus being conspecific was 0.05–0.5. *Liocranchia reinhardtii* was recognized as a single species with a probability of 0.95–1. *Leachia* cf. *atlantica* was recognized as a single species with a probability of 0.95–1. *Leachia lemur* and *Le. atlantica* were recognized as the same species with a probability of 0.95–1. The probability that all species of the genus *Leachia* are conspecific was 0.5–0-9. The probability of a single species within *E. inermis* was 0.95–1. The two *B. lyromma* sequences were each recognized as a single species with a probability of 0.95–1. *Helicocranchia navossae* spp. 1 and 2 were recognized as conspecific with a probability of 0.95–1. The two *Ga. armata* sequences were each recognized as one species with probabilities of 0.95–1. The probability of both *Ga. armata* sequences being conspecific was 0.5–0.9. *Teuthowenia megalops* and *T. pellucida* were each recognized as species with a probability of 0.95–1, while the probability of the two of them being conspecific was 0.5–0.9. The probability that the two *Gr. bonplandi* each represented a single species was 0.95-1. The probability *Gr. bonplandi* sequences were conspecific was 0.5–0.9. *Abraliopsis morisii*, *Abraliopsis* sp. 2 and *Abraliopsis* sp. were each recognized as a single species with probabilities of 0.95–1. The probability that *Ab. morisii* and *Abraliopsis* sp. 2 are the same species was

0.5–0.9. The probability that all members of *Abraliopsis* are conspecific was 0.05–0.5. *Mastigoteuthis agassizii* was recognized as a single species with a probability of 0.95–1. Species of the family Pyroteuthidae were each recognized as a single species with a probability of 0.95–1. *Pterygoteuthis gemmata*, *Pt. giardi* and *Pt. hoylei* were recognized as conspecific with a probability of 0.5–0.9. The probability that the whole family is conspecific was 0.05–0.5.

Intra- and interspecific p-*distances*

Intraspecific *p*-distances of *cox1* ranged from 0.9 to 2.4 % (Table S2). Although in most cases distances were low (<1 %), six species intraspecific ranges were larger than 1%: *Ancistrocheirus* sp. 3 (0.6–1.5 %), *Le.* cf. *atlantica* (0.0–1.3 %), *T. pellucida* (0.0–2.1 %), *T. megalops* (0.0–1.9 %), *Gr. bonplandi* (0.0–2.4 %) and *M. agassizii* (0.0–2.0 %). *Ancistrocheirus* sp. 3, *T. pellucida*, *T. megalops*, *Gr. bonplandi* and *M. agassizii* were identified as more than a single species for at least one species delimitation method over the *cox1* databases (Fig. 1). Interspecific *cox1 p*-distances (Table S3) were on average 13.3 % (range 0.9–18.6 %). Thus, for the full *cox1* matrix, an overlap exists among the largest intraspecific and the lowest interspecific distances. Distances among *Chtenopteryx* spp. ranged from 2.5 to 13.9 %. Distances among *Ancistrocheirus* spp. were 9.0–13.0 %. Distances among *Leachia* spp. were 3.0–5.3 %. Distances among *Teuthowenia* spp. were 2.3–4.2 %. Distances among *Helicocranchia* spp. were 1.0–13.7 %; the lowest among them were between *H. navossae* spp. 1 and 2 (1.0 %) and between *Helicocranchia* sp. and *H. navossae* spp. 1 and 2 (2.1–2.4 %). It is worth noting that bPTP recovered the three clades as different species, TCS and mGMYC recovered *Helicocranchia* sp. as a different species, while ASAP and sGMYC recovered the three lineages as a single species. Distances among *Galiteuthis* spp. ranged from 3.4 to 6.9 %. Distances among *Abraliopsis* spp. were 6.0–12.9 %. The distance between *M. agassizii* and *M.* cf. *dentata* was 0.9 %. Distances among Pyroteuthidae spp. ranged from 1.3 to 16.7 %. Despite the fact that the distance between *Py. margaritifera* and *Py.* aff. *margaritifera* is low (1.3 %), bPTP, ASAP and sGMYC recovered them as different species. Intraspecific distances in both cases were minimal, as the *Py. margaritifera* haplotypes were identical and the two *Py.* aff. *margaritifera* haplotypes had a 0.3 divergence. *Pterygoteuthis gemmata* and *Pt.* cf. *gemmata* had a distance of 5.0 % and were recovered as different species by all species delimitation analyses.

Table S2. Intraspecific *p*-distances of *cox1* as percentage (%). Abbreviation: N/A, not applicable.

Species	Mean (%)	Range (%)	(n)
Chtenopteryx canariensis	N/A	N/A	1
Chtenopteryx cf. *canariensis*	0.3	0.0–0.7	5
Chtenopteryx sicula	0.0	0.0–0.2	20
Chtenopteryx cf. *sicula* sp. 2	N/A	N/A	1
Chtenopteryx cf. *sicula* sp. 3	N/A	N/A	1
Chtenopteryx cf. *sicula* sp. 4	0.2	0.0–0.2	4
Chtenopteryx sp. KER1	N/A	N/A	1
Chtenopteryx sp. KER2	0.0	0.0–0.0	3
Chtenopteryx sp. C	N/A	N/A	1
Ancistrocheirus sp. 1	0.0	0.0–0.3	11
Ancistrocheirus sp. 2	0.2	0.2–0.5	3
Ancistrocheirus sp. 3	0.4	0.6–1.5	3
Leachia cf. *atlantica*	0.6	0.0–1.3	13
Leachia lemur	0.2	0.0–0.5	6
Leachia separata	0.0	0.0–0.0	11
Liocranchia reinhardtii	0.2	0.0–0.5	22
Liocranchia valdiviae	N/A	N/A	1
Bathothauma lyromma	0.0	0.0–0.2	4
Egea inermis	0.1	0.0–0.2	4
Teuthowenia pellucida	0.5	0.0–2.1	3
Teuthowenia megalops	0.0	0.0–1.9	5
Teuthowenia maculata	0.0	0.0–0.3	9
Helicocranchia navossae sp. 1	0.2	0.0–0.5	9
Helicocranchia navossae sp. 2	0.2	0.0–0.3	3
Helicocranchia pffefferi sp. 1	N/A	N/A	1
Helicocranchia pffefferi sp. 2	0.2	0.0–0.6	6
Helicocranchia pffefferi sp. 3	N/A	N/A	1
Helicocranchia sp.	N/A	N/A	1
Helicranchia sp. KER	0.0	N/A	2
Galiteuthis armata	0.3	0.0–0.7	8
Galiteuthis sp. 2	0.3	N/A	2
Galiteuthis sp. KER	N/A	N/A	1
Galiteuthis sp. 3	0.0	0.0–0.2	4
Grimalditeuthis bonplandi	0.4	0.0–2.4	5
Abraliopsis morisii	0.3	0.0–0.9	8
Abraliopsis sp. 1	0.2	0.0–0.5	7
Abraliopsis sp. 2	N/A	N/A	1
Abraliopsis sp. 3	0.0	0.0–0.2	5
Abraliopsis atlantica	0.0	0.0–0.2	3
Abraliopsis tui	0.0	0.0–0.2	16
Mastigoteuthis agassizii	0.4	0.0–2.0	29
Mastigoteuthis cf. *dentata*	0.0	0.0-0.0	11
Mastigoteuthis psychrophila	0.3	0.0–0.4	10
Pyroteuthis margaritifera	0.0	0.0-0.0	36
Pyroteuthis aff. *margaritifera*	0.3	N/A	2
Pyroteuthis serrata	0.0	0.0-0.0	6
Pyroteuthis addolux	N/A	N/A	1
Pyroteuthis RJ-2009 / *Pterygioteuthis giardi*	0.3	N/A	2
Pterygioteuthis gemmata	0.3	0.0–0.8	25
Pterygioteuthis cf. *gemmata*	0.3	0.0–0.5	5
Pterygioteuthis giardi / *Pterygioteuthis hoylei*	0.0	0.0–0.0	10
Pterygioteuthis microlampas	0.0	N/A	2
Pterygioteuthis giardi	N/A	N/A	1

Table S3. Interspecific mean *p*-distance percentages (%) of *cox1* between oceanic squid lineages.

	1	2	3	4	5	6	7	8	9	10	11	12	13	14	15	16	17	18	19	20	21	22	23	24	25	26	27	28	29	30	31	32	33	34	35	36	37	38	39	40	41	42	43	44	45	46	47	48	49	50	51	52
Chtenopteryx canariensis [1]																																																				
Chtenopteryx cf. *canariensis* [2]	9.7																																																			
Chtenopteryx sicula [3]	7.9	10.2																																																	
Chtenopteryx cf. *sicula* sp. 2 [4]	7.6	11.3	7.1																																																
Chtenopteryx cf. *sicula* sp. 3 [5]	9.9	13.9	9.1	2.8																																															
Chtenopteryx cf. *sicula* sp. 4 [6]	8.0	10.6	8.5	7.9	9.9																																														
Chtenopteryx sp. KER1 [7]	5.6	9.3	5.9	5.9	7.9	7.1																																													
Chtenopteryx sp. KER2 [8]	7.9	11.3	7.9	7.4	9.9	6.8	6.5																																												
Chtenopteryx sp. C [9]	7.0	11.0	6.5	6.5	9.1	5.9	6.2	2.5																																											
Ancistrocheirus sp. 1 [10]	15.2	15.6	12.7	14.7	15.6	14.3	14.2	14.4	13.9																																										
Ancistrocheirus sp. 2 [11]	15.1	15.7	15.1	15.1	16.8	14.3	15.4	16.2	14.5	12.6																																									
Ancistrocheirus sp. 3 [12]	15.4	16.1	12.6	15.7	17.4	15.8	14.3	14.5	14.5	9.5	13.0																																								
Leachia cf. *atlantica* [13]	15.7	14.8	14.1	15.4	17.4	15.0	16.1	15.6	16.1	16.0	16.5	14.2																																							
Leachia lemur [14]	16.1	15.6	13.8	16.9	18.6	15.8	16.9	16.4	16.4	15.9	16.9	14.3	3.0																																						
Leachia separata [15]	16.7	14.4	13.3	16.1	18.1	15.0	16.7	16.7	15.6	17.3	15.1	15.6	5.3	5.3																																					
Liocranchia reinhardtii [16]	11.4	11.9	11.9	13.6	15.6	13.9	13.0	13.9	13.6	16.7	15.9	16.2	14.4	15.5	14.1																																				
Liocranchia valdiviae [17]	10.8	12.5	11.3	13.0	14.4	11.9	11.9	12.2	11.9	13.0	15.7	12.6	13.1	13.4	14.2	12.2																																			
Bathothauma lyromma [18]	13.9	13.0	12.5	13.9	15.6	15.4	13.6	15.0	15.0	17.0	17.4	16.0	15.4	16.8	16.4	11.9	11.0																																		
Egea inermis [19]	15.2	16.4	12.4	13.2	16.1	13.1	14.7	12.7	13.2	14.7	16.8	15.6	16.6	16.9	16.6	13.3	13.1	12.3																																	
Teuthowenia pellucida [20]	14.2	13.1	13.8	14.4	16.6	14.2	13.8	12.9	13.5	15.2	15.4	13.1	16.1	16.7	17.3	13.9	12.7	10.1	12.7																																
Teuthowenia megalops [21]	12.5	11.9	12.7	13.3	15.6	12.5	12.7	12.2	12.5	14.7	14.8	13.9	14.9	15.6	15.9	11.6	11.3	7.9	11.8	3.3																															
Teuthowenia maculata [22]	12.5	10.8	12.2	13.3	15.3	12.7	12.2	11.3	12.5	15.3	15.1	14.4	14.7	15.3	16.4	11.6	10.8	9.1	12.8	4.2	2.3																														
Helicocranchia navossae sp. 1 [23]	14.3	12.1	15.2	14.5	15.9	15.4	13.8	16.4	15.5	14.4	15.2	14.9	15.2	15.0	16.1	13.1	13.4	13.0	13.9	12.9	12.2	11.9																													
Helicocranchia navossae sp. 2 [24]	14.6	12.0	14.4	13.5	15.5	15.8	14.3	16.1	14.6	15.4	14.9	14.8	15.2	15.3	15.4	12.2	14.1	12.7	13.4	12.4	11.6	11.3	1.0																												
Helicocranchia pffefferi sp. 1 [25]	12.4	12.2	11.9	10.8	12.7	11.8	12.7	11.9	11.9	12.5	15.7	16.0	15.8	15.8	15.9	13.4	14.2	12.7	13.2	12.7	11.0	11.0	9.6	8.7																											
Helicocranchia pffefferi sp. 2 [26]	15.0	14.8	13.0	13.3	15.3	14.4	13.9	14.4	14.2	12.1	15.9	15.4	15.9	15.8	15.3	14.5	14.5	14.0	12.7	13.3	12.8	14.0	11.3	11.1	7.5																										
Helicocranchia pffefferi sp. 3 [27]	13.6	13.3	12.2	14.7	16.7	14.6	12.7	14.2	13.9	12.2	15.4	16.4	16.3	15.6	15.3	11.0	12.7	13.0	12.7	12.1	11.9	11.6	13.7	13.1	13.0	13.3																									
Helicocranchia sp. [28]	14.2	11.3	14.4	13.6	15.6	15.2	13.6	15.0	15.3	15.6	15.4	14.8	15.0	15.5	16.1	12.5	13.9	12.5	13.5	12.1	11.3	10.8	2.4	2.1	8.8	11.0	13.0																								
Helicranchia sp. KER [29]	11.8	11.3	12.2	11.6	13.6	11.2	11.9	11.9	12.2	12.5	16.0	16.7	15.3	15.0	15.0	13.3	13.9	12.5	13.0	13.5	11.3	11.3	9.6	9.4	2.8	7.6	12.2	8.8																							
Galiteuthis armata [30]	14.6	14.0	13.5	15.4	17.7	14.2	15.2	14.1	14.1	15.4	16.1	15.2	13.6	13.9	15.3	11.3	13.2	9.5	12.8	8.9	8.4	8.4	12.4	11.7	12.1	13.2	8.7	11.5	12.6																						
Galiteuthis sp. 2 [31]	13.0	13.3	12.5	15.0	16.4	13.2	13.9	14.4	14.7	13.9	15.4	15.0	13.8	13.6	14.7	10.2	11.0	9.3	11.8	8.1	7.6	7.6	12.7	12.7	10.8	11.3	7.4	12.2	10.2	4.4																					
Galiteuthis sp. KER [32]	12.6	14.2	13.3	15.3	17.0	13.2	15.3	15.3	15.0	14.2	16.5	16.1	14.7	14.2	14.2	11.9	11.9	10.5	11.5	9.8	9.9	10.8	11.9	12.4	12.2	12.0	8.8	12.2	12.2	6.9	5.4																				
Galiteuthis sp. 3 [33]	14.4	15.3	13.6	14.2	15.9	14.0	16.1	15.0	15.3	14.7	15.7	15.3	13.4	14.2	14.7	11.6	13.0	11.0	12.7	10.1	9.6	9.3	13.6	12.9	11.3	12.2	9.3	12.5	11.9	5.0	3.4	6.5																			
Grimalditeuthis bonplandi [34]	12.1	12.7	10.7	12.8	14.2	11.8	12.7	13.1	13.1	14.2	15.6	13.4	13.6	14.3	13.9	13.6	12.1	12.7	14.6	14.9	12.5	13.1	16.1	16.1	13.9	16.7	12.7	16.1	13.7	13.4	12.2	13.9	13.2																		
Abraliopsis morisii [35]	10.5	12.9	13.2	13.5	14.9	13.6	11.8	13.5	12.6	14.6	16.7	14.9	18.1	18.5	18.2	15.2	14.9	13.9	13.6	14.1	12.8	14.5	14.2	14.1	14.5	16.7	13.9	14.5	14.8	12.7	13.3	13.3	15.3	14.6																	
Abraliopsis sp. 1 [36]	12.9	12.4	13.0	13.0	14.7	13.6	13.6	13.9	13.9	17.0	16.0	16.3	16.4	16.5	16.6	16.3	14.8	15.6	15.5	16.5	15.5	15.6	14.2	14.3	13.9	15.5	16.1	14.1	13.9	15.8	14.2	15.3	15.3	13.9	12.8																
Abraliopsis sp. 2 [37]	9.7	13.3	11.6	13.0	14.7	12.7	11.3	11.9	11.3	14.4	16.0	14.4	16.5	16.7	16.4	14.4	12.7	14.7	13.2	14.8	12.7	13.6	14.8	14.6	14.4	17.0	12.5	14.4	14.7	13.7	13.0	13.6	14.2	13.4	6.0	10.7															
Abraliopsis sp. 3 [38]	11.3	12.7	12.2	10.8	13.0	12.7	11.9	10.5	10.5	14.7	14.0	14.0	15.4	15.8	16.4	13.8	12.5	15.3	14.7	16.1	14.4	14.4	13.9	13.5	13.3	15.5	14.4	12.7	13.0	14.0	14.4	14.7	15.0	14.1	11.5	10.0	9.6														

	1	2	3	4	5	6	7	8	9	10	11	12	13	14	15	16	17	18	19	20	21	22	23	24	25	26	27	28	29	30	31	32	33	34	35	36	37	38	39	40	41	42	43	44	45	46	47	48	49	50	51	52
Abraliopsis atlantica [39]	14.4	13.9	13.6	13.9	15.6	15.3	13.9	14.7	14.7	17.8	16.5	14.6	16.1	16.9	16.1	17.5	14.7	15.9	16.4	16.9	15.0	15.3	13.7	13.2	15.3	15.3	17.0	13.6	15.6	16.3	15.9	16.4	16.4	14.4	12.9	5.1	11.6	10.5														
Abraliopsis tui [40]	10.8	11.3	13.6	11.6	13.0	12.6	12.2	11.9	11.3	12.7	15.4	14.3	17.0	18.1	17.6	13.3	13.6	13.6	13.5	14.4	11.9	13.0	12.7	12.7	11.6	15.3	12.5	12.7	12.2	12.0	12.2	11.3	13.0	12.5	6.6	11.6	6.2	8.8	11.6													
Mastigoteuthis agassizii [41]	12.2	12.6	11.1	12.5	14.5	13.6	11.0	13.1	12.2	15.1	13.2	14.8	16.4	15.9	15.5	11.2	13.6	13.2	13.9	14.0	13.0	12.7	14.6	14.3	13.7	14.3	13.1	14.7	13.4	13.9	13.1	14.6	14.8	12.1	16.2	16.1	15.6	12.4	16.3	14.0												
Mastigoteuthis cf. *dentata* [42]	12.2	12.5	10.5	12.7	14.7	13.9	11.0	13.3	12.5	15.3	13.8	14.8	16.1	15.9	15.3	10.8	13.9	12.7	13.5	13.8	12.7	12.5	14.7	14.4	13.9	14.4	13.0	14.7	13.6	13.5	12.7	14.2	14.4	11.7	16.3	16.4	15.3	12.5	16.1	13.9	0.9											
Mastigoteuthis psychrophila [43]	12.9	13.0	10.7	13.5	14.9	14.4	11.9	13.8	12.9	14.4	14.5	13.9	14.4	14.4	14.9	11.3	12.9	10.8	14.3	12.5	11.9	11.9	13.2	13.4	14.4	15.0	13.7	13.8	14.6	12.9	11.9	11.7	13.8	11.1	16.5	14.5	15.3	14.1	14.6	13.2	5.4	4.8										
Pyroteuthis margaritifera [44]	10.7	13.0	10.2	10.8	12.5	10.5	11.0	10.8	10.5	12.2	15.4	13.9	13.8	13.6	15.0	12.4	10.2	14.2	12.1	11.2	10.2	10.5	12.1	12.7	10.8	11.9	10.8	12.7	9.6	10.7	8.8	9.9	10.5	12.1	12.1	13.1	11.3	11.6	14.2	11.0	14.2	14.4	13.5									
Pyroteuthis aff. *margaritifera* [45]	10.7	13.0	10.2	10.8	12.5	10.5	11.0	10.8	10.5	11.9	15.7	13.6	13.7	13.6	15.0	12.1	10.2	14.0	12.1	11.0	10.1	10.2	11.8	12.5	10.5	11.6	10.8	12.5	9.3	10.7	8.8	9.9	10.5	12.3	12.2	13.2	11.5	11.8	14.3	11.2	13.9	14.2	13.2	1.3								
Pyroteuthis serrata [46]	10.4	11.6	10.2	11.0	13.3	9.6	11.0	11.0	10.8	13.9	15.4	14.2	13.4	13.6	14.7	12.7	10.5	13.9	12.4	11.5	10.8	10.5	12.2	12.7	12.2	13.3	12.2	12.5	11.0	11.5	10.2	11.0	11.6	12.7	11.5	12.2	10.5	10.8	13.3	11.6	14.0	13.9	12.9	4.0	3.5							
Pyroteuthis addolux [47]	12.5	15.6	13.6	13.3	15.9	14.7	13.3	12.2	12.7	14.4	13.4	12.4	14.8	15.9	16.1	15.3	11.3	13.6	13.5	14.9	13.6	14.4	16.8	16.9	16.4	15.5	15.9	16.4	15.6	15.1	15.3	16.1	15.9	14.1	13.2	15.9	11.9	11.6	15.0	12.2	16.4	16.1	15.9	12.5	12.3	13.3						
Pyroteuthis RJ-2009 / *Pterygioteuthis giardi* [48]	12.5	15.6	13.3	12.2	13.9	13.7	14.1	12.2	12.2	14.2	14.6	14.5	15.0	15.7	15.7	14.5	12.0	14.5	14.2	15.5	14.8	14.5	15.2	15.0	13.6	17.0	15.1	15.0	14.2	15.8	14.2	15.1	14.8	11.7	13.0	13.2	9.7	8.8	15.1	10.0	16.3	16.2	15.6	11.4	11.3	10.9	8.6					
Pterygioteuthis gemmata [49]	12.6	14.7	13.0	12.9	14.6	12.2	12.9	12.9	12.4	13.5	12.0	12.0	14.9	15.6	14.1	13.7	10.4	13.9	14.7	14.9	13.5	13.8	17.1	17.2	15.3	16.9	14.7	16.8	15.6	14.9	13.7	14.2	14.8	12.5	11.5	12.9	10.4	9.9	13.8	10.8	14.2	14.3	13.5	11.3	10.8	10.7	6.7	7.7				
Pterygioteuthis cf. *gemmata* [50]	13.6	14.7	13.0	14.4	15.9	13.9	13.6	13.3	12.7	12.7	13.7	11.5	16.0	15.6	16.7	15.6	11.3	14.7	15.2	14.1	13.0	14.2	15.5	15.8	14.7	16.2	15.3	15.3	16.1	13.2	13.0	13.9	15.0	14.1	10.4	14.0	10.5	10.5	15.3	10.5	16.1	16.1	14.5	11.3	11.2	12.2	6.5	8.3	5.0			
Pterygioteuthis giardi / *Pterygioteuthis hoylei* [51]	12.4	14.3	11.8	14.3	16.0	15.7	12.9	13.7	13.7	15.2	17.8	16.9	15.7	15.7	16.3	14.8	13.7	13.2	14.0	14.2	12.9	12.6	14.2	14.2	14.6	14.3	10.9	14.3	14.6	10.5	10.6	11.5	12.0	14.0	10.4	13.8	10.6	12.3	14.3	12.3	15.6	15.4	14.9	10.1	10.1	11.5	13.2	12.1	12.5	10.9		
Pterygioteuthis microlampas [52]	14.0	15.0	14.4	16.1	17.8	16.0	15.6	15.0	15.0	14.7	17.4	16.7	16.7	16.1	17.3	14.7	15.3	14.4	15.5	14.4	13.6	13.3	14.4	14.9	14.7	14.3	11.3	14.2	15.3	12.1	12.5	12.7	13.0	13.9	14.0	16.1	14.2	14.7	15.6	15.0	15.1	14.7	13.4	12.2	11.9	12.7	16.7	15.7	14.5	14.7	6.9	
Pterygioteuthis giardi [53]	13.9	16.1	13.6	14.7	16.4	15.6	14.4	14.4	15.0	14.7	14.5	12.3	14.1	15.0	15.3	14.2	11.0	13.3	12.7	13.8	12.5	13.0	16.2	15.8	14.7	13.9	14.4	15.3	14.4	14.3	14.2	14.7	15.3	13.9	14.2	15.7	12.5	11.3	15.0	13.3	14.7	14.7	14.8	12.7	12.6	12.7	4.8	8.6	8.1	7.9	12.9	15.6

12S intraspecific *p*-distances ranged from 0.0 to 8.4 % (Table S4). However, all the species with intraspecific lineages over 1.8 % were identified as more than a single species for two or more species delimitation methods (Fig. S2). The single exception is *Pt. gemmata*, which only the mGMYC identified it as four different species instead of a single one. Interspecific *p*-distances (Table S5) had a mean value of 18.1 % and ranged from 0 % to 32.4 %. If the distance among *H. novossae* lineages is removed, the lowest interspecific distance is 1.6 % between *Ab. morisii* and *Abraliopsis* sp. 2 and 1.7 % between *T. megalops* and *T. maculata*. Thus, for 12S, an overlap exists among the largest intraspecific and the lowest interspecific distances. Distance among *Chtenopteryx* spp. ranged from 9.8 to 13.9 %. Distance between *Ancistrocheirus* spp. 1 and 2 was 11.6 %. Distance among *Teuthowenia* spp. was 1.7–4.8 %. Distance between *Ga. armata* and *Galiteuthis* sp. 2 was 4.1 %. Distances among *Abraliopsis* spp. was 1.6–11.4 %. The distance between *Pt. gemmata* and *Pterygioteuthis* sp. 2 was 23.2 %.

Table S4. Intraspecific *p*-distances of 12S as percentage (%). Abbreviation: N/A, not applicable.

Species	Mean (%)	Range (%)	n
Chtenopteryx canariensis	N/A	N/A	1
Chtenopteryx cf. *canariensis*	N/A	N/A	1
Chtenopteryx sicula	0.13	0–0.6	13
Ancistrocheirus sp. 1	0.74	0–6.1	12
Ancistrocheirus sp. 2	N/A	N/A	1
Leachia cf. *atlantica*	0.60	0.0–1.8	10
Liocranchia reinhardtii	0.07	0.0–3.9	23
Bathothauma lyromma	0.0	0.0–0.0	3
Teuthowenia pellucida	N/A	N/A	1
Teuthowenia megalops	N/A	N/A	1
Teuthowenia maculata	0.18	0.0–1.2	9
Helicocranchia navossae sp. 1	N/A	N/A	1
Helicocranchia navossae sp. 2	0.0	0.0–0.0	3
Galiteuthis armata	1.63	0.0–0.0	4
Galiteuthis sp. 2	N/A	N/A	1
Grimalditeuthis bonplandi	0.54	0.0–4.5	3
Abraliopsis morisii	0.0	0.0–0.6	3
Abraliopsis sp. 1	3.66	0.0–8.4	4
Abraliopsis sp. 2	N/A	N/A	1
Mastigoteuthis agassizii	0.0	0.0–0.6	3
Pyroteuthis margaritifera	0.0	0.0–0.0	7
Pterygioteuthis gemmata	0.42	0.0–6.8	24
Pterygioteuthis sp. 2	0.0	N/A	2

Table S5. Interspecific mean *p*-distance percentages (%) of 12S between oceanic squid lineages.

	1	2	3	4	5	6	7	8	9	10	11	12	13	14	15	16	17	18	19	20	21	22
***Chtenopteryx canariensis* [1]**																						
***Chtenopteryx* cf. *canariensis* [2]**	13.8																					
***Chtenopteryx sicula* [3]**	9.8	13.9																				
***Ancistrocheirus* sp. 1 [4]**	22.1	18.9	20.6																			
***Ancistrocheirus* sp. 2 [5]**	17.1	15.4	16.3	11.6																		
***Leachia* cf. *atlantica* [6]**	32.4	29.2	27.5	25.7	23.7																	
***Liocranchia reinhardtii* [7]**	25.2	19.5	22.9	18.9	12.2	19.4																
***Bathothauma lyromma* [8]**	23.6	22.8	23.6	19.6	14.6	18.8	12.2															
***Teuthowenia pellucida* [9]**	22.0	21.1	20.4	17.2	15.4	18.8	13.9	6.5														
***Teuthowenia megalops* [10]**	19.5	20.3	19.6	15.6	13.8	20.4	13.9	7.3	3.3													
***Teuthowenia maculata* [11]**	21.2	21.9	21.1	17.2	15.4	21.9	15.5	9.0	4.8	1.7												
***Helicocranchia navossae* sp. 1 [12]**	23.6	21.1	23.6	19.7	15.4	17.0	9.8	10.6	13.8	13.8	15.5											
***Helicocranchia navossae* sp. 2 [13]**	23.6	21.1	23.6	19.7	15.4	17.0	9.8	10.6	13.8	13.8	15.5	0.0										
***Galiteuthis armata* [14]**	20.3	24.4	21.2	19.6	14.6	21.2	12.2	7.3	8.9	8.1	9.8	11.4	11.4									
***Galiteuthis* sp. 2 [15]**	22.8	26.0	22.0	20.5	15.4	20.4	14.7	9.8	11.4	8.9	10.7	13.0	13.0	4.1								
***Grimalditeuthis bonplandi* [16]**	20.6	21.4	16.6	17.5	8.4	19.1	15.8	12.5	14.1	14.9	16.5	15.7	15.7	15.7	14.1							
***Abraliopsis morisii* [17]**	26.0	24.4	26.1	19.9	15.4	21.1	15.5	14.6	16.3	17.9	19.5	15.4	15.4	18.7	17.9	12.5						
***Abraliopsis* sp. 1 [18]**	26.4	26.6	28.5	22.7	15.2	22.6	15.5	18.3	17.3	18.1	19.7	21.5	21.5	18.9	20.1	15.7	9.8					
***Abraliopsis* sp. 2 [19]**	27.6	26.0	26.1	21.5	17.1	21.1	17.1	14.6	16.3	17.9	19.5	17.1	17.1	18.7	17.9	12.5	1.6	11.4				
***Mastigoteuthis agassizii* [20]**	21.1	20.3	17.1	16.5	10.6	18.8	17.9	14.6	15.4	15.4	17.1	17.9	17.9	17.9	16.3	3.8	13.8	17.9	13.8			
***Pyroteuthis margaritifera* [21]**	22.0	18.7	17.9	18.9	11.4	22.8	15.5	13.0	14.6	13.8	13.8	16.3	16.3	14.6	14.6	9.2	16.3	19.1	16.3	10.6		
***Pterygioteuthis gemmata* [22]**	31.3	30.5	30.5	25.6	24.8	26.5	25.6	24.0	23.2	24.8	26.4	24.8	24.8	24.0	24.8	24.3	22.3	26.4	22.3	24.0	24.8	
***Pterygioteuthis* sp. 2 [23]**	27.6	22.8	25.3	26.0	19.5	26.9	22.0	24.4	23.6	22.8	24.3	22.0	22.0	23.6	25.2	20.6	19.5	19.5	20.3	20.3	17.9	23.2

Intraspecific representation in the mitoPCG is low, with four species having two sequences (Table S6). Intraspecific *p*-distances ranged from 0.0 to 0.9 %. Interspecific distances (Table S7) range from 2 to 25.5 %, with a mean value of 19.8 %. *Chtenopteryx* spp. interspecific distances ranged from 10.3 to 13.9 %. *Ancistrocheirus* spp. distances ranged from 16.0 to 16.7 %. *Teuthowenia pellucida* and *T. megalops* had 8.7 % divergence. *Helicocranchia navossae* mitogenomes had a comparatively lower distance value of 2 %. The *p*-distance between *Ab. morisii* and *Abraliopsis* sp. 2 was 10.5 %.

Table S6. Intraspecific *p*-distances of mitoPCG as percentage (%). Abbreviation: N/A, not applicable.

Species	Mean	n
Chtenopteryx canariensis	N/A	1
Chtenopteryx cf. *canariensis*	N/A	1
Chtenopteryx sicula	N/A	1
Chtenopteryx cf. *sicula* sp. 4	N/A	1
Ancistrocheirus sp. 1	N/A	1
Ancistrocheirus sp. 2	N/A	1
Ancistrocheirus sp. 3	N/A	1
Leachia cf. *atlantica*	N/A	1
Liocranchia reinhardtii	N/A	1
Egea inermis	0.0	2
Bathothauma lyromma	0.03	2
Teuthowenia megalops	N/A	1
Teuthowenia pellucida	N/A	1
Helicocranchia navossae sp. 1	N/A	1
Helicocranchia navossae sp. 2	N/A	1
Galiteuthis armata	N/A	1
Grimalditeuthis bonplandi	N/A	1
Abraliopsis morisii	N/A	1
Abraliopsis sp. 2	N/A	1
Mastigoteuthis agassizii	0.92	2
Pyroteuthis margaritifera	0.0	2

Table S7. Interspecific mean *p*-distance percentages (%) of mitoPCG between oceanic squid lineages.

	1	2	3	4	5	6	7	8	9	10	11	12	13	14	15	16	17	18	19	20
***Chtenopteryx canariensis* [1]**																				
***Chtenopteryx* cf. *canariensis* [2]**	13.7																			
***Chtenopteryx sicula* [3]**	10.9	12.9																		
***Chtenopteryx* cf. *sicula* sp. 4 [4]**	10.6	13.9	10.3																	
***Ancistrocheirus* sp. 1 [5]**	20.7	20.3	19.7	20.4																
***Ancistrocheirus* sp. 2 [6]**	21.7	21.8	21.2	21.3	16.0															
***Ancistrocheirus* sp. 3 [7]**	22.0	22.1	20.5	21.1	16.1	16.7														
***Leachia* cf. *atlantica* [8]**	22.5	22.4	21.9	21.9	23.0	23.8	24.4													
***Liocranchia reinhardtii* [9]**	21.2	21.5	20.5	21.4	22.0	23.1	23.4	22.1												
***Egea inermis* [10]**	17.9	17.6	17.1	18.6	21.4	21.2	21.9	22.0	19.8											
***Bathothauma lyromma* [11]**	18.8	19.6	18.1	19.1	21.8	21.9	22.7	23.2	20.9	16.5										
***Teuthowenia pellucida* [12]**	19.5	19.1	17.8	19.5	20.8	21.8	22.5	22.7	20.7	14.8	18.5									
***Teuthowenia megalops* [13]**	18.9	18.5	18.0	18.7	20.6	21.4	21.9	21.6	20.0	14.6	18.2	8.7								
***Helicocranchia navossae* sp. 1 [14]**	20.1	19.3	18.9	20.2	21.1	21.4	22.5	22.1	19.8	16.7	16.7	16.9	17.2							
***Helicocranchia navossae* sp. 2 [15]**	20.2	19.5	18.8	20.5	21.5	21.3	22.2	22.2	19.6	16.6	16.6	16.9	16.9	2.0						
***Galiteuthis armata* [16]**	18.7	18.5	17.7	18.8	21.0	20.7	21.7	21.9	19.6	13.3	17.1	15.0	15.0	16.9	16.8					
***Grimalditeuthis bonplandi* [17]**	19.4	19.3	19.0	19.6	21.0	20.7	21.7	22.6	20.5	18.9	19.2	20.3	20.0	18.7	18.4	18.6				
***Abraliopsis morisii* [18]**	21.6	22.4	20.8	22.2	22.4	21.9	21.7	25.1	22.7	22.1	22.9	22.5	22.0	22.5	22.5	21.4	22.5			
***Abraliopsis* sp. 2 [19]**	21.4	22.1	20.9	21.7	22.3	21.9	21.5	25.5	22.8	21.8	22.7	23.0	22.1	22.7	22.8	21.7	22.3	10.5		
***Mastigoteuthis agassizii* [20]**	18.2	18.5	17.7	18.2	20.3	20.9	20.8	22.0	20.7	18.1	19.8	18.3	18.8	19.1	19.4	18.5	17.0	22.5	22.3	
***Pyroteuthis margaritifera* [21]**	19.0	19.1	17.5	17.8	20.4	21.6	22.1	22.4	19.6	19.1	20.1	19.1	18.6	19.6	19.9	18.5	19.9	20.1	20.6	19.6

For 18S, intraspecific distances ranged from 0.0 to 1.2 % (Table S8). The only species which showed intralineage variation in *p*-distance for this marker were *C. sicula* (0.0–0.07 %) and *B. lyromma* (1.2 %). Interspecific mean distance (Table S9) across the full dataset was 2.1 % (range 0.0–5.1 %). Distance among *Chtenopteryx* spp. was 0.0–0.2 %. Distance among *Ancistrocheirus* spp. was 0.0–0.1 %. Distance among *Leachia* spp. was 0.1–0.2 %; note that *Le.* cf. *atlantica* and *Le. atlantica* are provided separately as *Leachia atlantica* is paraphyletic including *Le. lemur* (Fig. S4) and there is no *cox1*, 12S or mitoPCG sequence available for that specimen to place its phylogenetic position using a more variable marker. Distance between *T. megalops* and *T. pellucida* was 0.1 %. Distance between *H. novossae* spp. 1 and 2 was 0.0 %. Distance among *Abraliopsis* spp. was 0.1–4.5 %. Distance among *Pterygioteuthis* spp. was 0.4–1.8 %. As a rule of thumb it can be said that 18S intraspecific *p*-distances are lower than interspecific distances and range from 0 to 0.07 %. Interspecific distances usually are higher than 0.1 %. The single exception with higher intraspecific distances than those found among different species is *B. lyromma*, with 1.2 % distance.

Table S8. Intraspecific *p*-distances of 18S as percentage (%). Abbreviation: N/A, not applicable.

**Species**	**Mean**	**Range (%)**	**n**
Chtenopteryx canariensis	N/A	N/A	1
Chtenopteryx cf. *canariensis*	N/A	N/A	1
Chtenopteryx sicula	0.04	0.0–0.07	3
Chtenopteryx cf. *sicula* sp. 4	N/A	N/A	1
Ancistrocheirus sp. 1	N/A	N/A	1
Ancistrocheirus sp. 2	N/A	N/A	1
Ancistrocheirus sp. 3	N/A	N/A	1
Leachia cf. *atlantica*	N/A	N/A	1
Leachia atlantica	N/A	N/A	1
Leachia lemur	N/A	N/A	1
Liocranchia reinhardtii	N/A	N/A	1
Bathothauma lyromma	1.2	N/A	2
Egea inermis	0	N/A	2
Teuthowenia megalops	N/A	N/A	1
Teuthowenia pellucida	N/A	N/A	1
Helicocranchia navossae sp. 1	N/A	N/A	1
Helicocranchia navossae sp. 2	N/A	N/A	1
Galiteuthis armata	0	N/A	2
Abraliopsis morisii	0	N/A	2
Abraliopsis sp.	N/A	N/A	1
Abraliopsis sp. 2	N/A	N/A	1
Mastigoteuthis agassizii	0	0.0–0.0	3
Grimalditeuthis bonplandi	0	N/A	2
Pyroteuthis margaritifera	0	0.0–0.0	3
Pterygioteuthis giardi	N/A	N/A	1
Pterygioteuthis gemmata	N/A	N/A	1
Pterygioteuthis microlampas	N/A	N/A	1
Pterygioteuthis hoylei	N/A	N/A	1

Table S9. Interspecific mean *p*-distance percentages (%) of 18S between oceanic squid lineages.

	1	2	3	4	5	6	7	8	9	10	11	12	13	14	15	16	17	18	19	20	21	22	23	24	25	26	27
***Chtenopteryx canariensis* [1]**																											
***Chtenopteryx* cf. *canariensis* [2]**	0.1																										
***Chtenopteryx sicula* [3]**	0.2	0.0																									
***Chtenopteryx* cf. *sicula* sp. 4 [4]**	0.0	0.1	0.2																								
***Ancistrocheirus* sp. 1 [5]**	1.8	1.9	1.9	1.8																							
***Ancistrocheirus* sp. 3 [6]**	1.8	1.9	1.9	1.8	0.0																						
***Ancistrocheirus* sp. 2 [7]**	1.7	1.8	1.8	1.7	0.1	0.1																					
***Leachia* cf. *atlantica* [8]**	2.3	2.3	2.4	2.3	1.2	1.2	1.1																				
***Leachia atlantica* [9]**	2.4	2.5	2.5	2.4	1.4	1.4	1.2	0.1																			
***Leachia lemur* [10]**	2.5	2.5	2.6	2.5	1.4	1.4	1.3	0.2	0.1																		
***Liocranchia reinhardtii* [11]**	2.5	2.6	2.6	2.5	2.3	2.3	2.2	2.3	2.4	2.5																	
***Bathothauma lyromma* [12]**	1.4	1.5	1.5	1.4	1.0	1.0	0.8	1.2	1.4	1.4	2.1																
***Egea inermis* [13]**	1.4	1.4	1.5	1.4	1.0	1.0	0.8	1.3	1.4	1.5	2.3	0.3															
***Teuthowenia megalops* [14]**	1.5	1.6	1.6	1.5	1.1	1.1	1.0	1.1	1.2	1.3	2.1	0.1	0.3														
***Teuthowenia pellucida* [15]**	1.4	1.5	1.5	1.4	1.0	1.0	0.9	1.2	1.3	1.4	2.0	0.1	0.3	0.1													
***Helicocranchia navossae* sp. 1 [16]**	1.7	1.7	1.7	1.7	1.0	1.0	1.0	1.1	1.2	1.3	2.4	0.7	0.7	0.7	0.6												
***Helicocranchia navossae* sp. 2 [17]**	1.7	1.7	1.7	1.7	1.0	1.0	1.0	1.1	1.2	1.3	2.4	0.7	0.7	0.7	0.6	0.0											
***Galiteuthis armata* [18]**	1.4	1.5	1.5	1.4	1.2	1.2	1.1	1.4	1.5	1.6	2.1	0.4	0.6	0.4	0.3	0.8	0.8										
***Abraliopsis morisii* [19]**	3.7	3.7	3.7	3.7	3.9	3.9	3.9	3.5	3.5	3.6	3.3	3.8	4.0	3.7	3.8	3.9	3.9	3.7									
***Abraliopsis* sp. [20]**	4.3	4.3	4.4	4.3	3.9	3.9	3.8	3.7	3.7	3.7	5.1	4.3	4.1	4.2	4.3	4.0	4.0	4.3	4.5								
***Abraliopsis* sp. 2 [21]**	3.7	3.7	3.7	3.7	3.9	3.9	3.9	3.5	3.5	3.6	3.3	3.8	4.0	3.7	3.8	3.9	3.9	3.7	0.1	4.5							
***Mastigoteuthis agassizii* [22]**	1.9	1.9	2.0	1.9	0.6	0.6	0.6	1.2	1.3	1.4	2.2	0.9	1.0	1.0	1.0	1.2	1.2	1.0	4.0	4.0	4.0						
***Grimalditeuthis bonplandi* [23]**	2.3	2.2	2.2	2.3	0.8	0.8	0.9	1.6	1.7	1.8	2.5	1.2	1.3	1.4	1.3	1.4	1.4	1.4	4.1	4.3	4.1	0.5					
***Pyroteuthis margaritifera* [24]**	2.1	2.2	2.2	2.1	0.9	0.9	0.8	1.7	1.9	1.9	2.5	1.4	1.4	1.5	1.4	1.4	1.4	1.2	3.9	4.1	3.9	0.6	0.8				
***Pterygioteuthis giardi* [25]**	2.4	2.5	2.5	2.4	1.6	1.6	1.5	2.4	2.5	2.6	3.1	2.1	2.1	2.3	2.2	2.3	2.3	2.0	3.9	3.5	3.9	1.4	1.8	1.2			
***Pterygioteuthis gemmata* [26]**	2.6	2.8	2.8	2.6	1.9	1.9	1.9	2.8	3.0	3.0	3.3	2.4	2.4	2.5	2.5	2.5	2.5	2.3	3.9	3.9	3.9	1.8	2.0	1.3	0.4		
***Pterygioteuthis microlampas* [27]**	2.5	2.5	2.5	2.5	1.3	1.3	1.2	1.9	2.0	2.1	2.8	1.9	1.8	1.9	1.9	2.0	2.0	1.7	4.2	4.0	4.2	1.0	1.2	0.7	1.5	1.8	
***Pterygioteuthis hoylei* [28]**	2.5	2.6	2.6	2.5	1.7	1.7	1.7	2.5	2.7	2.8	3.2	2.3	2.3	2.4	2.3	2.4	2.4	2.1	4.1	3.5	4.1	1.6	1.9	1.4	0.1	0.6	1.7

Species distribution ranges

Chtenopteryx *spp.*

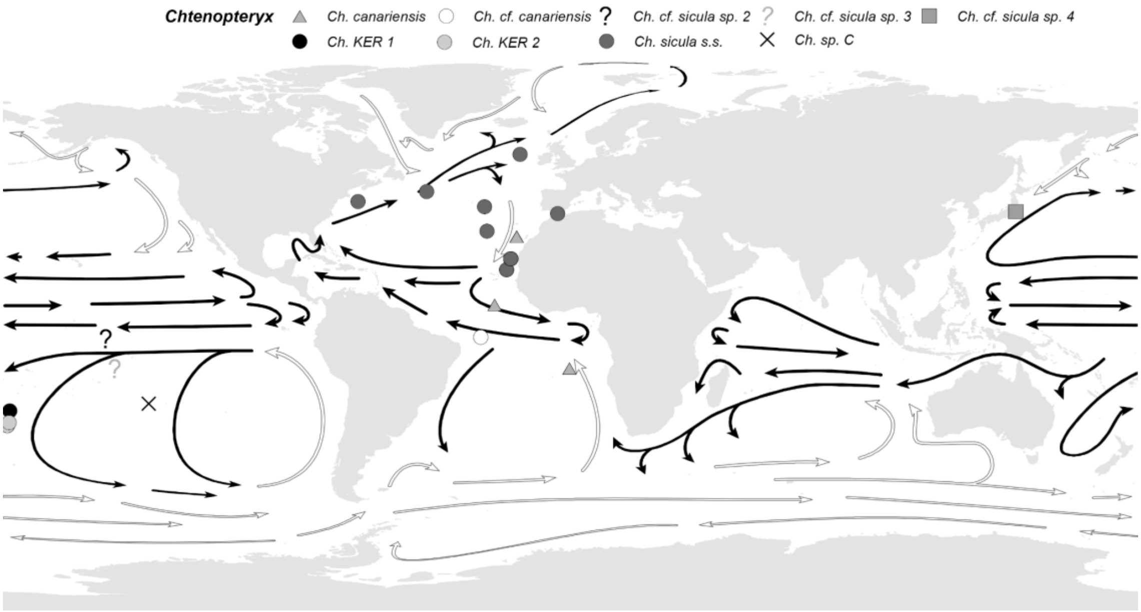

Fig. S9. Distribution of *Chtenopteryx* spp. studied in this work according to the major clades found in this study (Table S1). Black and white arrows depict major world currents. When possible, specific coordinates were used to plot each geographic occurrence. Question marks represent points that were ambiguous in the publication and/or the GenBank record.

Ancistrocheirus *spp.*

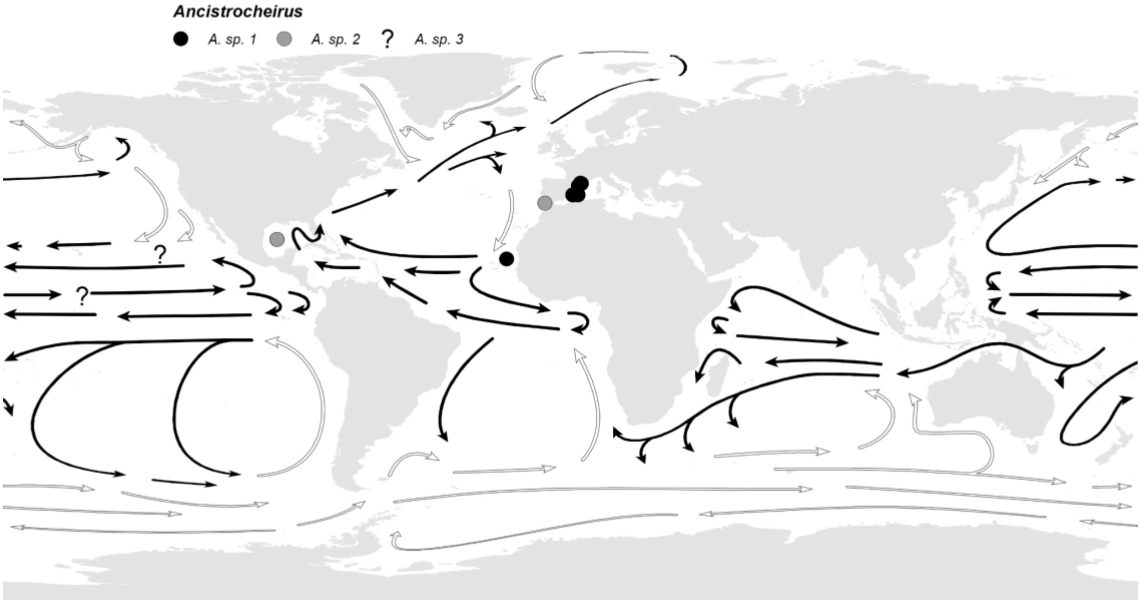

Fig. S10. Distribution of *Ancistrocheirus* spp. studied in this work according to the major clades found in this study (Table S1). Black and white arrows depict major world currents. When possible, specific coordinates were used to plot each geographic occurrence. Question marks represent points that were ambiguous in the publication and/or the GenBank record.

Leachia *spp.*

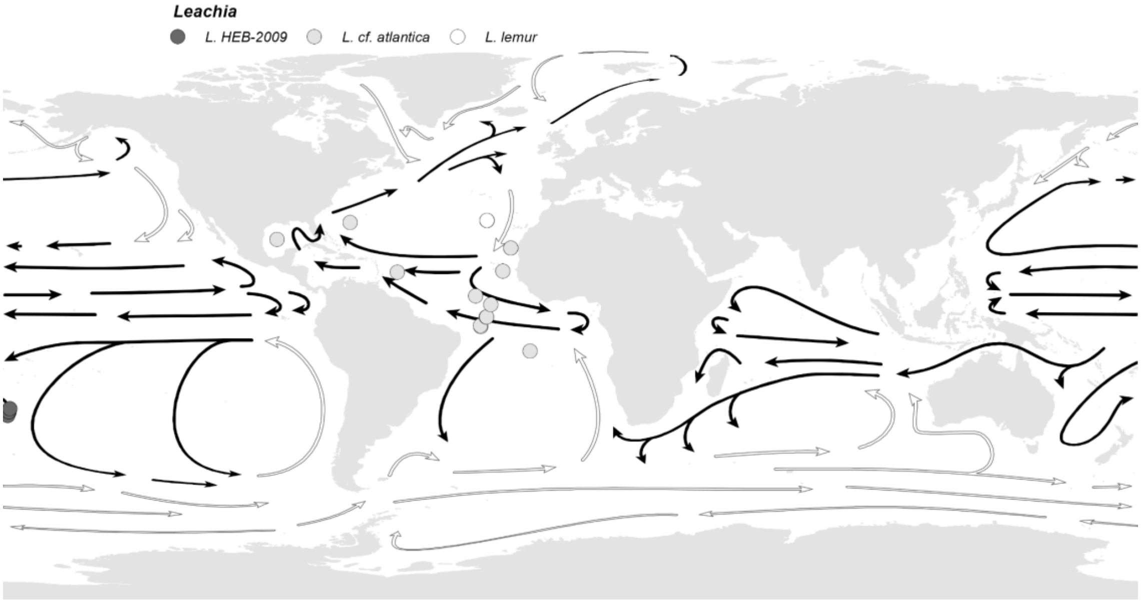

Fig. S11. Distribution of *Leachia* spp. studied in this work according to the major clades found in this study (Table S1). Black and white arrows depict major world currents. When possible, specific coordinates were used to plot each geographic occurrence.

Liocranchia *spp.*

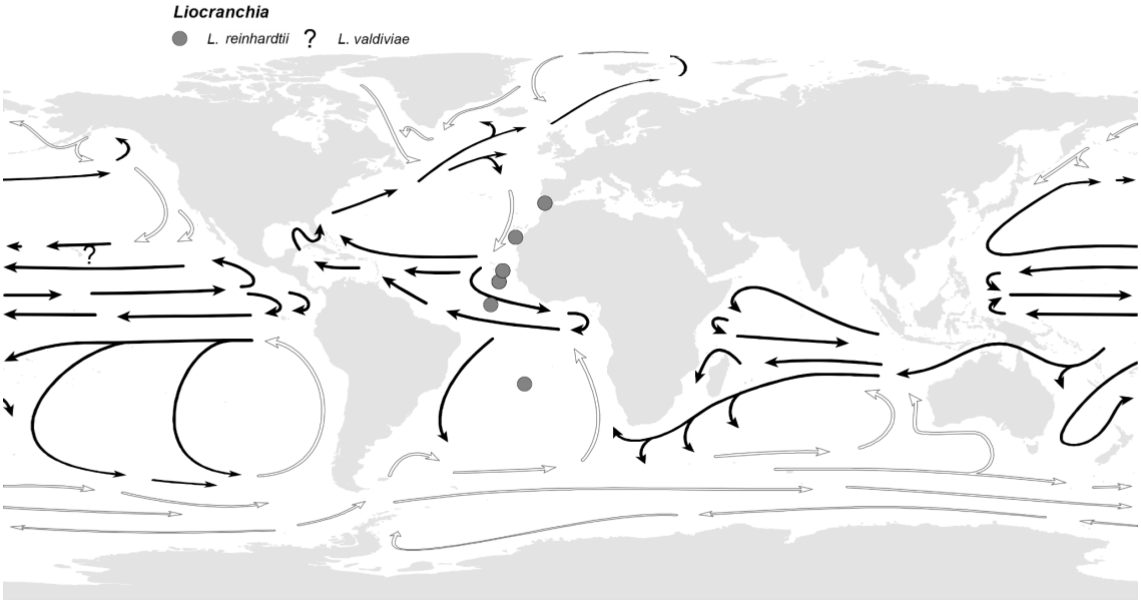

Fig. S12. Distribution of *Liocranchia* spp. studied in this work according to the major clades found in this study (Table S1). Black and white arrows depict major world currents. When possible, specific coordinates were used to plot each geographic occurrence. Question marks represent points that were ambiguous in the publication and/or the GenBank record.

Egea inermis *and* Bathothauma lyromma

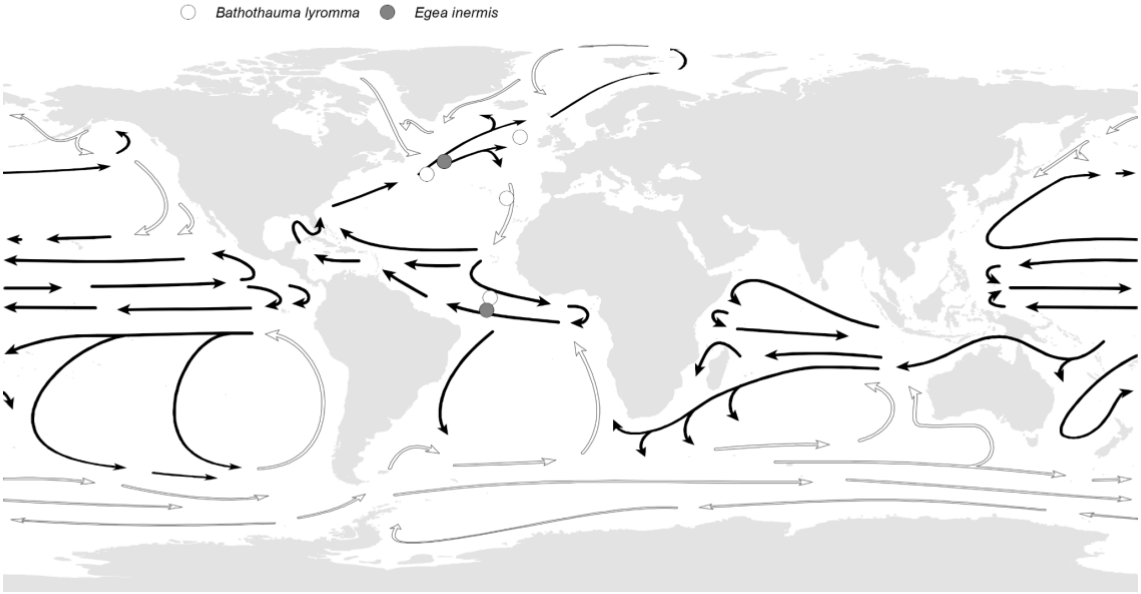

Fig. S13. Distribution of the studied sequences of *Bathothauma lyromma* and *Egea inermis* (Table S1). Black and white arrows depict major world currents. When possible, specific coordinates were used to plot each geographic occurrence.

Teuthowenia *spp.*

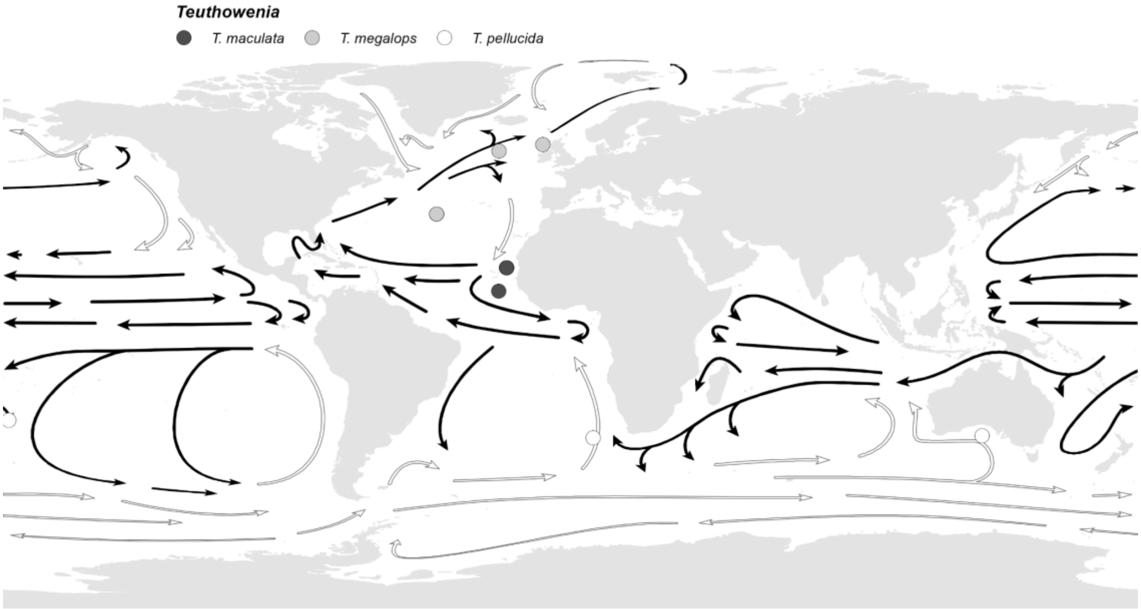

Fig. S14. Distribution of *Teuthowenia* spp. according to the major clades found in this study (Table S1). Black and white arrows depict major world currents. When possible, specific coordinates were used to plot each geographic occurrence.

Heliconcranchia *spp.*

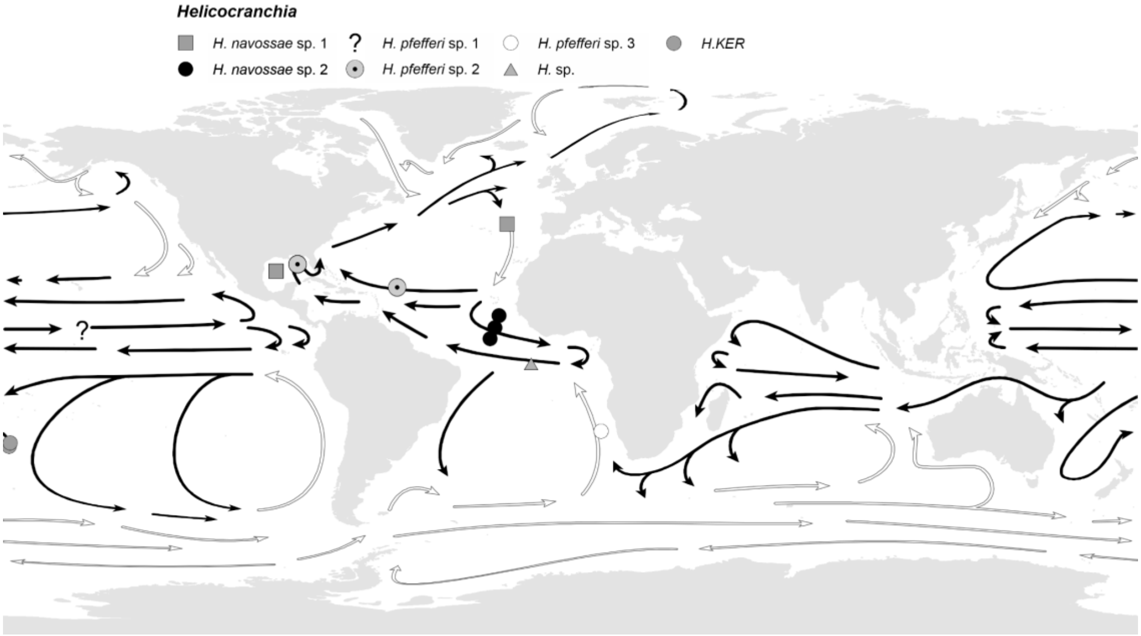

Fig. S15. Distribution of *Helicocranchia* spp. studied in this work according to the major clades found in this study (Table S1). Black and white arrows depict major world currents. When possible, specific coordinates were used to plot each geographic occurrence. Question marks represent points that were ambiguous in the publication and/or the GenBank record.

Galiteuthis *spp.*

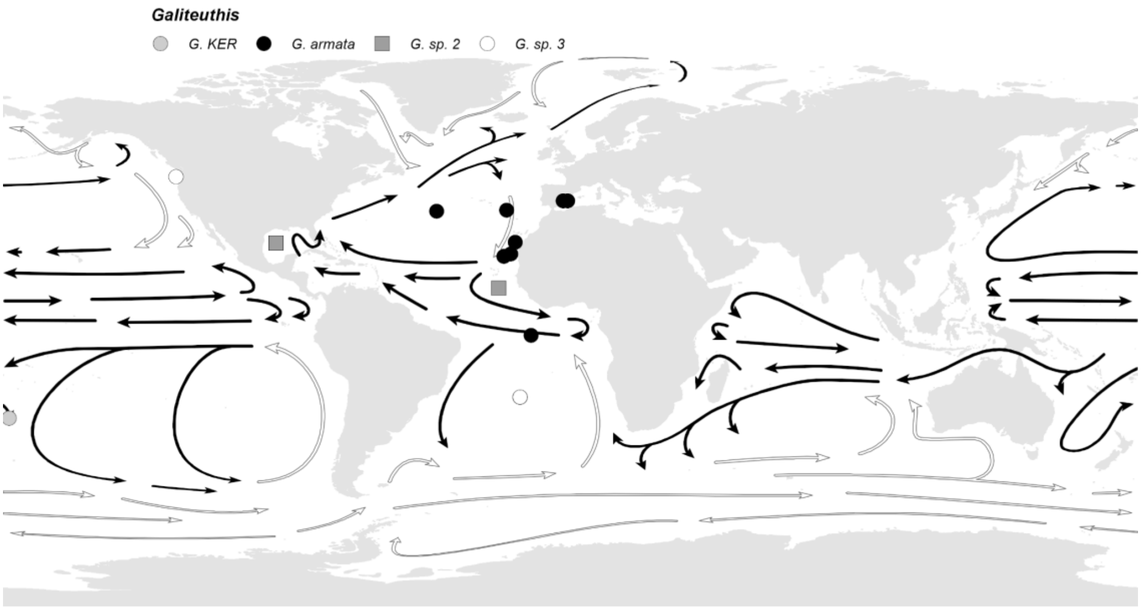

Fig. S16. Distribution of *Galiteuthis* spp. studied in this work according to the major clades found in this study (Table S1). Black and white arrows depict major world currents. When possible, specific coordinates were used to plot each geographic occurrence.

Chiroteuthidae and Mastigoteuthidae

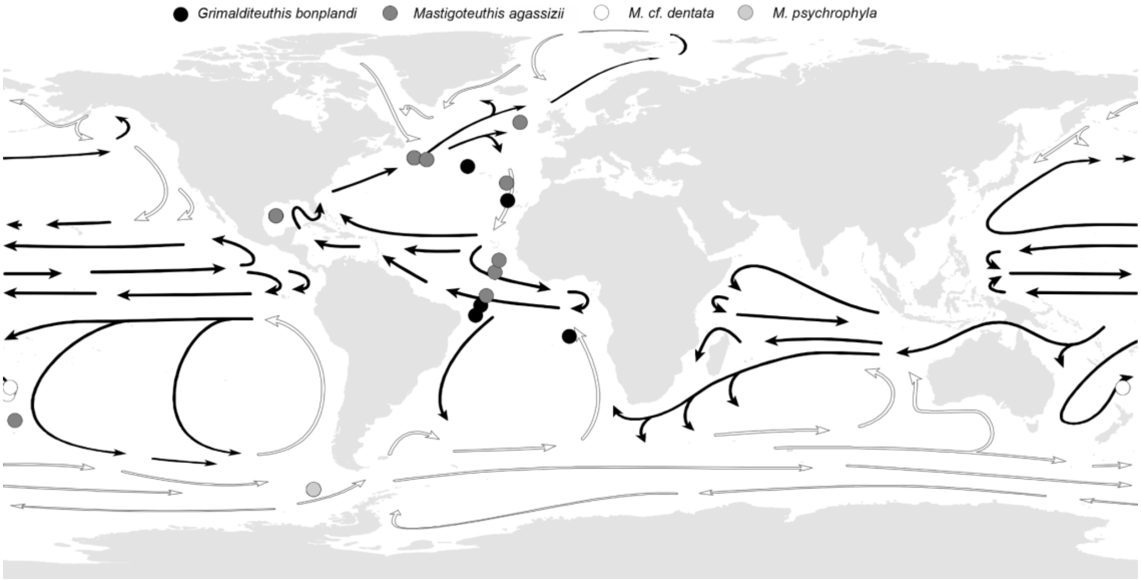

Fig. S17. Distribution of *Grimalditeuthis bonplandi* and *Mastigoteuthis* spp. studied in this work (Table S1). Black and white arrows depict major world currents. When possible, specific coordinates were used to plot each geographic occurrence.

Abraliopsis *spp.*

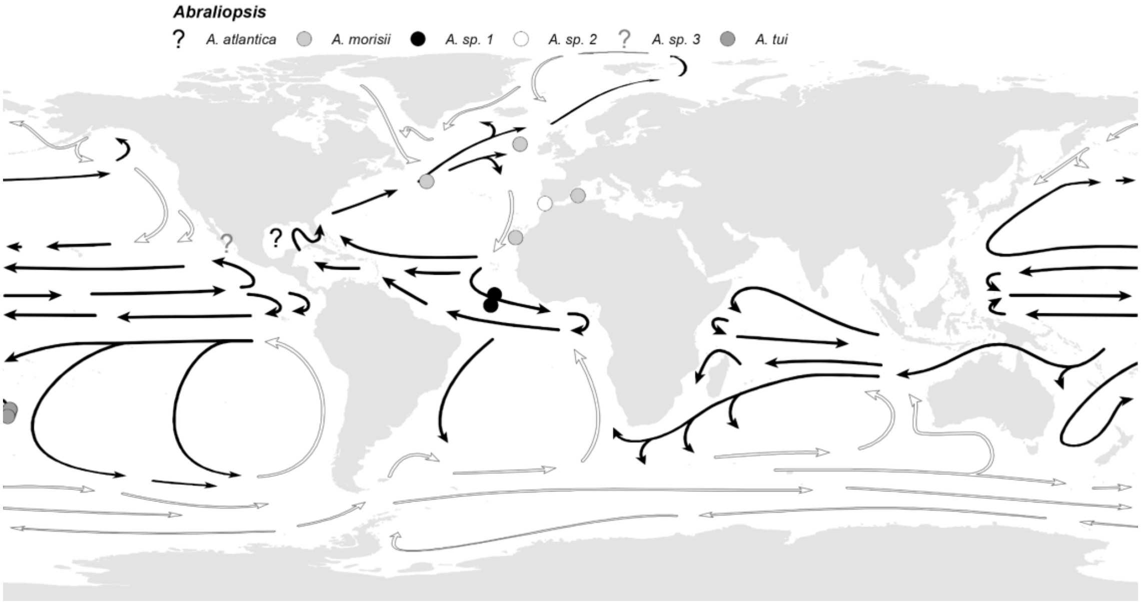

Fig. S18. Distribution of *Abraliosis* spp. studied in this work according to the major clades found in this study (Table S1). Black and white arrows depict major world currents. When possible, specific coordinates were used to plot each geographic occurrence. Question marks represent points that were ambiguous in the publication and/or the GenBank record.

Pterygioteuthis *spp.*

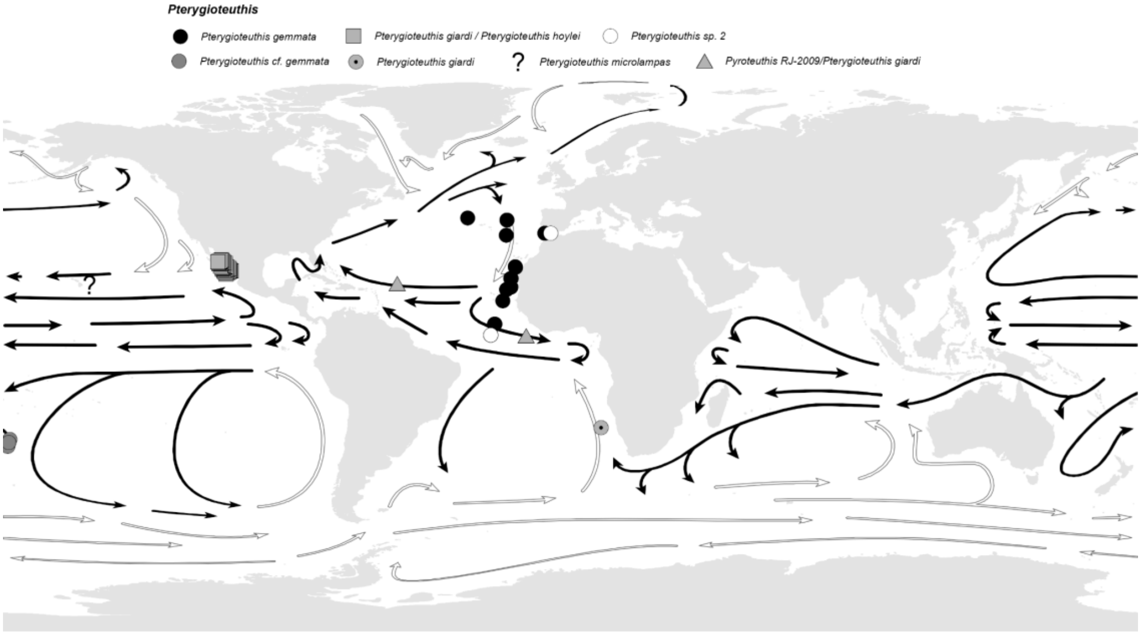

Fig. S19. Distribution of *Pterygioteuthis* spp. studied in this work according to the major clades found in this study (Table S1). Black and white arrows depict major world currents. When possible, specific coordinates were used to plot each geographic occurrence. Question marks represent points that were ambiguous in the publication and/or the GenBank record.

Pyroteuthis *spp.*

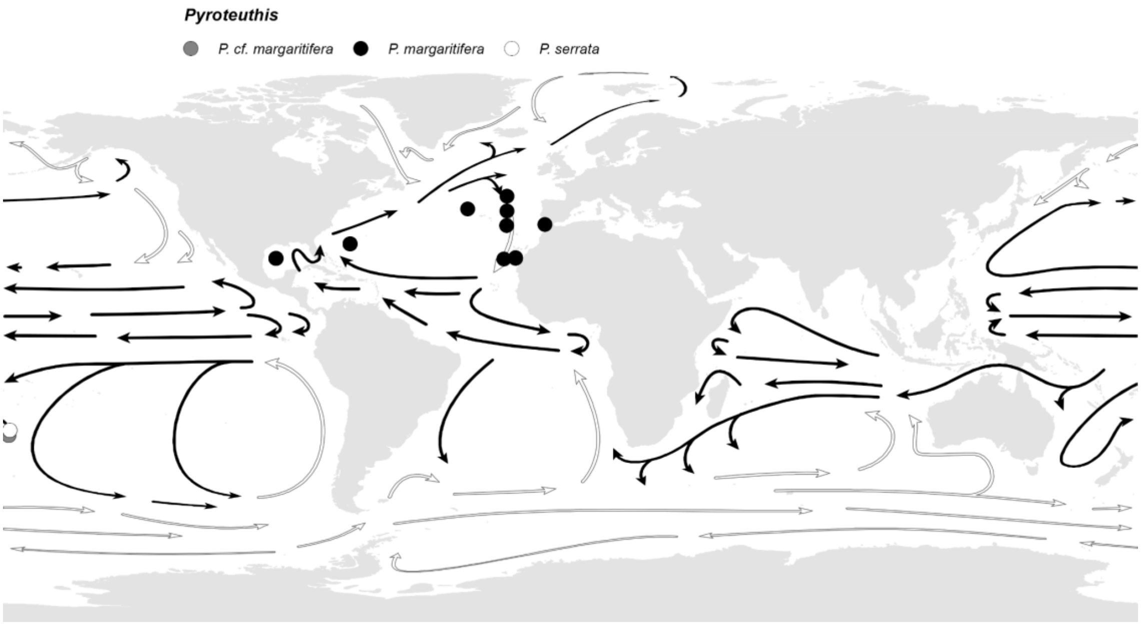

Fig. S20. Distribution of *Pyroteuthis* spp. studied in this work according to the major clades found in this study (Table S1). Black and white arrows depict major world currents. When possible, specific coordinates were used to plot each geographic occurrence.

Supplementary references including those from Table S1

Anderson FE. 2000. Phylogeny and historical biogeography of the loliginid squids (Mollusca: Cephalopoda) based on mitochondrial DNA sequence data. Mol Phylogen Evol 15: 191–214

Andrews S. 2010. FastQC: a quality control tool for high throughput sequence data. http:// (www.bioinformatics.babraham.ac.uk/projects/fastqc)

Appellöf A. 1890. Teuthologische Beitrage I: *Chtenopteryx* n.g., *Veranya sicula* Krohn, *Calliteuthis* Verrill. Bergens Mus Aarsberetning 1889: 1–34

Bernt M, Donath A, Jühling F, Externbrink F, Florentz C, Fritzsch G, Pütz J, Middendorf M, Stadler PF (2013) MITOS: improved de novo metazoan mitochondrial genome annotation. Mol Phylogenet Evol 69: 313–319

Berry SS. 1913. Some new Hawaiian cephalopods. Proc US Natl Mus 45: 563–566

Berry SS. 1920. Preliminary diagnosis of new cephalopods from the western Atlantic. Proc US Natl Mus 58: 293–300

Bolstad KSR, Perez JAA, Strugnell JM, Vidal EAG. 2015. Cranchiids of the South Atlantic Mid-Oceanic Ridge: results from the first southern MAR-ECO expedition. J Nat His 49: 21–24

Bouckaert R, Xie D. 2017. Standard nucleotide substitution models v1.0.1. (https:doi.org/10.5281/zenodo.995740)

Bouckaert R, Vaughan TG, Barido-Sottani J, Duchêne S, Fourment M, Gavryushkina A, Heled J, Jones G, Kühnert D, De Maio N et al. 2019. BEAST 2.5: an advanced software platform for Bayesian evolutionary analysis. PLoS Comput Biol 15: 1–28

Braid HE, Bolstad KSR. 2019. Cephalopod biodiversity of the Kermadec Islands: implications for conservation and some future taxonomic priorities. Invertebr Syst 33: 402–425

Braid HE, McBride PD, Bolstad KSR. 2014. Molecular phylogenetic analysis of the squid family Mastigoteuthidae (Mollusca, Cephalopoda) based on three mitochondrial genes. Hydrobiologia 725: 145–164

Bucklin A, Ortman BD, Jennings RM, Nigro LM, Sweetman CJ, Copley NJ, Sutton T, Wiebe PH. 2010. A "Rosetta Stone" for metazoan zooplankton: DNA barcode analysis of species diversity of the Sargasso Sea (Northwest Atlantic Ocean). Deep Sea Res II 57: 24–26

Carlini DB, Graves JE. 1999. Phylogenetic analysis of cytochrome C oxidase I sequences to determine higher–level relationships within the coleoid cephalopods. Bull Mar Sci 64: 57–76

Carrasco SA, Meerhoff E, Yannicelly B, Ibáñez CM. 2019. First records and descriptions of early life stages of cephalopods from Rapa Nui (Easter Island) and the nearby Apolo Seamount. Pac Sci: 163–175

Chun C. 1906. System der Cranchien. Zoologischer Anzeiger 31: 82–86

Chun C. 1908. Uber Cephalopoden der Deutschen Tiefsee–Expedition. Zoologischer Anzeiger 33(2: 86–89

Chun C. 1910. Die Cephalopoden. Oegopsida. Wissenschaftliche Eregebnisse der Deutschen Tiefsee Expedition auf dem Dampfer "Valdivia" 1898–1899. 18: 1–401

Clement M, Posada D, Crandall K. 2000. TCS: a computer program to estimate gene genealogies. Mol Ecol 9: 1657–1660

De Queiroz K. 2007. Species concepts and species delimitation. Syst Biol 56: 879–886

De Silva-Dávila R, Hochberg HG, Lindgren AR Franco-Gordo MC. 2013. Paralarval development, abundance, and distribution of *Pterygioteuthis hoylei* (Cephalopoda: Oegopsida: Pyroteuthidae) in the Gulf of California, México. Moll Res 33 50–64

Degner E. 1925. Cephalopoda. Report on the Danish Oceanographical Expeditions 1908 to 1910 to the Mediterranean and Adjacent Seas 2: 1–94

Dierckxsens N, Mardulyn P, Smits G. 2016. NOVOPlasty: de novo assembly of organelle genomes from whole genome data. Nucleic Acids Res 45: e18

Dodsworth S. 2015. Genome skimming for next-generation biodiversity analysis. Trends Plant Sci 20: 525–527

Drummond AJ, Ho SYW, Phillips MJ, Rambaut A. 2006. Relaxed phylogenetics and dating with confidence. PLoS Biol 4: 699–710

Drummond AJ, Nicholls GK, Rodrigo AG, Solomon W. 2002. Estimating mutation parameters, population history and genealogy simultaneously from temporally spaced sequence data. Genetics 161: 1307–1320

Escánez A, Roura Á, Riera R, González ÁF, Guerra Á. 2018. New data on the systematics of comb-fin squids *Chtenopteryx* spp. (Cephalopoda: Chtenopterygidae) from the Canary Islands. Zool Stu 57: 40

Evans AB, Bolstad KSR. 2023. Diversity of the squid genus *Leachia* (Oegopsida: Cranchiidae) in the Pacific Ocean. Mar Biol 170: 72

Fernández-Álvarez FÁ, Taite M, Vecchione M, Villanueva R, Allcock AL. 2022. A phylogenomic look into the systematics of oceanic squids (order Oegopsida). Zool J Linn Soc 194: 1212–1235

Fischer H. 1896. Note préliminaire sur le *Pterygioteuthis giardi*, céphalopode nouveau recueilli dans le cours de l'Expédition scientifique du Talisman (1883). J Conchyliol 43 :205–211

Folmer O, Black M, Hoeh W, Lutz R, Vrijenhoek R. 1994. DNA primers for amplification of mitochondrial cytochrome c oxidase subunit I from diverse metazoan invertebrates. Mol Mar Biol Biotechnol 3: 294–299

Fujisawa T, Barraclough TG. 2013. Delimiting species using single locus data and the generalized mixed yule coalescent approach: a revised method and evaluation on simulated data sets. Syst Biol 62: 707–724

Gray JE. 1849. Catalogue of the Mollusca in the Collection of the British Museum. Part I. Cephalopoda Artepedia. London. 164 pp.

Grimpe G. 1922. Systematische ubersicht der europaischen Cephalopoden. Sitzungsberichte der Naturforschenden Gesellschaft zu Leipzig 45: 36–52

Hoang DT, Chernomor O, von Haeseler A, Minh BQ, Vinh LS. 2018. UFBoot2: improving the ultrafast bootstrap approximation. Mol Biol Evol 35: 518–522

Hoyle WE. 1904. Reports on the Cephalopoda. Mem Mus Comp Zool Harv Coll 43: 1–72

Joubin L. 1896. Observations sur divers Céphalopodes. Premiere Note: Abraliopsis pfefferi (nov. gen. et spec.). Bulletin de la Societe Scientifique et Medicale de l'Ouest 5: 19–35

Joubin L. 1898a. Note sur une nouvelle famille de Céphalopodes. Ann Sci Nat Zool 6: 279–292

Joubin L. 1898b. Observations sur divers Céphalopodes. Quatrieme note: Grimalditeuthis richardi. Bull Soc Zool France 23: 101–113

Joubin L. 1933. Notes préliminaires sur les Céphalopodes des croisières du DANA (1921–1922), 4e Partie. Ann Inst Oceanogr 13: 1–49

Judkins H, Rose-Mann L, Lindgren A, Taite M, Bush S, Vecchione M. 2022. A newly discovered *Helicocranchia* species (Cephalopoda: Cranchiidae: Taoniinae) in the northern Gulf of Mexico. Bull Mar Sci 98: 419–430

Kalyaanamoorthy S, Minh BQ, Wong TKF, von Haeseler A, Jermiin LS. 2017. ModelFinder: fast model selection for accurate phylogenetic estimates. Nat Methods 14: 587–589

Kang X-X, Fernández-Álvarez FÁ, Alfaya JEF, Machordom A, Strand M, Sundberg P, Sun S-C. 2015. Species diversity of *Ramphogordius sanguineus/Lineus ruber*-like nemerteans (Nemertea: Heteronemertea) and geographic distribution of *R. sanguineus*. Zool Sci 32: 579–589

Lagesen K, Hallin P, Rødland EA, Stærfeldt HH, Rognes T, Ussery DW. 2007. RNAmmer: consistent and rapid annotation of ribosomal RNA genes. Nucleic Acids Res 35: 3100–3108

Leach WE. 1817. Synopsis of the orders, families, and genera of the class Cephalopoda. In: Leach WE, Nodder RP, editors. The Zoological Miscellany; being Descriptions of New or Interesting animals. London: McMillan. p. 137–141

Lesueur CA. 1821. Description of several new species of cuttlefish. J Acad Nat Sci Phila 2: 86–101

Lindgren AR. 2010. Molecular inference of phylogenetic relationships among Decapodiformes (Mollusca: Cephalopoda) with special focus on the squid Order Oegopsida. Mol Phylogen Evol 56: 77–90

Lindgren AR, Giribet G, Nishiguchi MK. 2004. A combined approach to the phylogeny of Cephalopoda (Mollusca). Cladistics 20: 454–486

Massy AL. 1907. Preliminary notice of new and remarkable cephalopods from the south-west coast of Ireland. Ann Mag Nat His 20: 377–384

Nesis KN. 1977. *Mastigoteuthis psychrophila* sp.n. (Cephalopoda, Mastigoteuthidae) from the Southern Ocean. Zool Zhurnal 56: 835–842

Nesis KN. 1982. Abridged key to the cephalopod molluscs of the world's oceans. Moscow: Light and Food Industry Publishing House. 385 pp.

Nguyen LT, Schmidt HA, von Haeseler A, Minh BQ. 2015. IQTREE: a fast and effective stochastic algorithm for estimating maximum-likelihood phylogenies. Mol Biol Evol 32: 268–274

Nishiguchi MK, Lopez JE, Boletzky Sv. 2004. Enlightenment of old ideas from new investigations: more questions regarding the evolution of bacteriogenic light organs in squids. Evol Dev 6: 41–49

Okonechnikov K, Golosova O, Fursov M, the UGENE team (2012). "Unipro UGENE: a unified bioinformatics toolkit". Bioinformatics 28: 1166–1667.

d'Orbigny A. 1834–1847. Mollusques. Voy Amer 5: 1–758

Passamaneck YJ, Schander C, Halanych KM. 2004. Investigation of molluscan phylogeny using large-subunit and small-subunit nuclear rRNA sequences. Mol Phylogen Evol 32, 25–38

Pfeffer G. 1884. Die Cephalopoden des Hamburger Naturhistorischen Museums. Abh Geb Naturw Hamburg 8, 1–30

Pfeffer G. 1912. Die Cephalopoden der Plankton-Expedition. Zugleich eine monographische übersicht der Oegopsiden Cephalopoden. Ergebnisse der Plankton-Expedition der Humboldt-stiftung 2: 1–815

Prosch, V. 1847. Nogle nye Cephalopoder, beskrevne og Anatomisk undersogte. Kongel Danske Vidensk Selsk Skr Naturvidensk Math 1: 53–72

Puillandre N, Brouillet S, Acha G. 2020. ASAP: assemble species by automatic partitioning. Mol Ecol Resour 21: 609–620

Rambaut A. 2010. FigTree v1.3.1. (http://tree.bio.ed.ac.uk/software/figtree/)

Rambaut A, Drummond AJ, Xie D, Baele G, Suchard MA. 2018. Posterior summarization in Bayesian phylogenetics using Tracer 1.7. Syst Biol 67: 901–904

Reid NM, Carstens BC. 2012. Phylogenetic estimation error can decrease the accuracy of species delimitation: a Bayesian implementation of the general mixed Yule-coalescent model. BMC Evol Biol 12 196

Riddell, D.J. 1985. The Enoploteuthidae (Cephalopoda: Oegopsida) of the New Zealand region. Fish Res Bull 27: 52

Rüppell, E. 1844. Intorno ad alcuni cefalopodi del mare di Messina: lettera del Dr. Eduardo Ruppell di Frankfort sul Meno al Prof. Anastasio Cocco. Giornale del Gabinetto Letterario di Messina 5: 129–135

Salcedo-Vargas MA, Guerrero-Kommritz J. 2000. Three new cephalopods from the Atlantic Ocean. Zoolog Inst und Zoolog Museum Hamburg 97: 31–44

Steenstrup, J. 1856. Hectoctyldannelsen hos Octopodslaegterne Argonauta og Tremoctopus, oplyst ved Iagttagelse af lignende Dannelser hos Blacksprutterne i Almindelighed.Kongelige Danske Videnskabernes Selskabs Skrifter, 5 Raekke. Naturvidensk Math 4: 185–216.

Strugnell J, Norman M, Jackson J, Drummond AJ, Cooper A. 2005. Molecular phylogeny of coleoid cephalopods (Mollusca: Cephalopoda) using a multigene approach; the effect of data

partitioning on resolving phylogenies in a Bayesian framework. Mol Phylogenet Evol 37: 426–441

Strugnell JM, Hall NE, Vecchione M, Fuchs D, Allcock AL. 2017. Whole mitochondrial genome of the ram's horn squid shines light on the phylogenetic position of the monotypic order Spirulida (Haeckel, 1896). Mol Phylogenet Evol 109: 296–301

Taite M, Vecchione M, Fennell S, Allcock LA. 2020. Paralarval and juvenile cephalopods within warm-core eddies in the North Atlantic. Bull Mar Sci 96 235–262

Tanner AR, Fuchs D, Winkelmann IE, Gilbert MTP, Pankey MS, Ribeiro ÂM, Kocot KM, Halanych KM, Oakley TH, da Fonseca RC et al. 2017. Molecular clocks indicate turnover and diversification of modern coleoid cephalopods during the Mesozoic Marine Revolution. Proc Royal Soc B 284: 20162818

Yokobori S, Fukuda N, Nakamura M, Aoyama T, Oshima T. 2004. Long-term conservation of six duplicated structural genes in cephalopod mitochondrial genomes. Mol Biol Evol 21: 2034–2046

Young RE. 1972. The systematics and areal distribution of pelagic cephalopods from the seas off Southern California. Washington (DC): Smithsonian Contributions to Zoology, 97. 159 pp.

Young RE, Lindgren A, Vecchione M. 2008. *Mastigoteuthis microlucens*, a new species of the squid family Mastigoteuthidae (Mollusca: Cephalopoda). Proc Biol Soc Washington 121: 276–282.

Verany JB. 1839. Memoire sur six nouvelles espèces de Céphalopodes trouves dans la Mediterranee a Nice. Memorie Accad Sci Torino 1: 91–98

Verany JB. 1851. Mollusques Méditerranéens observé décrits figurés et chromolithographiés d'après le vivant ouvrage dedie à SM le Roi Charles Albert. Gènes. 132 pp

Verrill AE. 1881a. The Cephalopods of the northeastern Coast of America. Part II. The smaller Cephalopods, including the "Squids" and the Octopi, with Other Allied Forms. Trans Conn Acad Arts Sci 5: 308–343

Verrill AE. 1881b. Report on the cephalopods, and some additional species dredged by the U.S. Fish Commission Steamer "Fish Hawk," during the season of 1880. Bull Mus Comp Zool 8: 99–116
